# Drought effect on plant biomass allocation: A meta‐analysis

**DOI:** 10.1002/ece3.3630

**Published:** 2017-11-12

**Authors:** Anwar Eziz, Zhengbing Yan, Di Tian, Wenxuan Han, Zhiyao Tang, Jingyun Fang

**Affiliations:** ^1^ Department of Ecology College of Urban and Environmental Sciences Peking University Beijing China; ^2^ Key Laboratory of Plant‐Soil Interactions Ministry of Education College of Resources and Environmental Sciences China Agricultural University Beijing China

**Keywords:** allometry, biomass allocation, biomass fraction, drought, life form, meta‐analysis

## Abstract

Drought is one of the abiotic stresses controlling plant function and ecological stability. In the context of climate change, drought is predicted to occur more frequently in the future. Despite numerous attempts to clarify the overall effects of drought stress on the growth and physiological processes of plants, a comprehensive evaluation on the impacts of drought stress on biomass allocation, especially on reproductive tissues, remains elusive. We conducted a meta‐analysis by synthesizing 164 published studies to elucidate patterns of plant biomass allocation in relation to drought stress. Results showed that drought significantly increased the fraction of root mass but decreased that of stem, leaf, and reproductive mass. Roots of herbaceous plants were more sensitive to drought than woody plants that reduced reproductive allocation more sharply than the former. Relative to herbaceous plants, drought had a more negative impact on leaf mass fraction of woody plants. Among the herbaceous plants, roots of annuals responded to drought stress more strongly than perennial herbs, but their reproductive allocation was less sensitive to drought than the perennial herbs. In addition, cultivated and wild plants seemed to respond to drought stress in a similar way. Drought stress did not change the scaling exponents of the allometric relationship between different plant tissues. These findings suggest that the allometric partitioning theory, rather than the optimal partitioning theory, better explains the drought‐induced changes in biomass allocation strategies.

## 1 INTRODUCTION

Drought is one of the major abiotic stresses that restrict terrestrial plant growth (Ferreira, De Lacerda, Costa, & Filho, 2015; Lambers, Chapin, & Pons, 2008). This negative impact is expected to be more pronounced in the future due to increasing drought frequency (Murray & Ebi, 2012). Therefore, it is urgent to understand the adaptation mechanism of plants to drought stress. Allocating resources to different structures or functions is the central concept of life history theory and determines the fitness and reproductive success of plants in a certain environment or in a community (Poorter, Remkes, & Lambers, 1990; Weiner, 2004). Among all the resources that have been proposed, biomass is likely to be the most feasible variable to quantify the resource allocation in plants (Bazzaz, Chiariello, Coley, & Pitelka, 1987; Harper & Ogden, 1970).

There are two ways of explaining biomass allocation of plants: the ratio‐based optimal partitioning theory (Bloom, Chapin, & Mooney, 1985) and the size‐dependent allometric partitioning theory (Niklas, 1993; Weiner, 1990; West, Brown, & Enquist, 1997). According to the optimal partitioning theory, plants allocate more proportional amount of resources (e.g., biomass) to the structure, by which the limiting resources (e.g., water, nutrients, or light) are captured, to optimize the plant performance (Bloom et al., 1985). For example, plants invest more biomass to root when water is scarce (Poorter et al., 2012). The concept of allometry was first introduced to life science by Huxley (1924), who found that relative growth rate of different organs is a power function of body size. As total annual growth is composed of growth of different modules (organs), this functional relation is also true for different parts of the plant and stable throughout the plant life history (Niklas, 1994, 2004). Evolutionary biologists suggested that this functional relationship between relative growth rates of different parts of an organism is the result of natural selection, which optimizes the fitness of organism (Egset et al., 2012; Voje, Hansen, Egset, Bolstad, & Pelabon, 2014; Weiner, 2004). Thus, researchers suggested that variation in resource allocation ratios is resulted from proportional relationship between relative growth rates of organs. (Enquist, West, Charnov, & Brown, 1999; Niklas, 1991; West et al., 1997). According to allometric partitioning theory, the single evolved allometric scaling can meet the environmental requirements that cause variation in biomass allocation strategy of plants explained by optimal partitioning theory (Müller, Schmid, & Weiner, 2000; Niklas, 1993; Weiner, 2004; West et al., 1997). Allometric partitioning theory has been validated in plants growing under various environmental gradients. For example, Bernacchi, Coleman, Bazzaz, and McConnaughay (2000) reported that allometric slopes between different parts of annual herbs are quite stable under CO_2_ enrichment. One recent meta‐analysis reported that the stable allometric scaling relationship exists between root mass and shoot mass of five different biomes across the globe under N enrichment (Peng & Yang, 2016).

Drought exerts strong impacts on biomass allocation. From the conventional ratio‐based perspective of allocation, the patterns of biomass allocation in relation to drought are still equivocal due to the sharp differences in the experimental conditions, treatment procedures, and plant materials among studies (Poorter et al., 2012; Wang, Taub, & Jablonski, 2015; Xie et al., 2016). For instance, Erice, Louahlia, Jose Irigoyen, Sanchez‐Diaz, and Avice (2010) reported that with the increased soil aridity, leaf dry mass fraction (LMF) of *Medicago sativa* decreased by 30% on average; stem mass fraction (SMF), on the other hand, increased by 4% on average. By contrast, Mao et al. (2012) found that increased drought enhanced LMF of *Setaria viridis* by about 30% on average. Through synthesizing multiple studies, Poorter et al. (2012) found that drought stress generally decreased LMF and SMF, and increased root mass fraction (RMF). However, to date, there is still a lack of knowledge regarding the changes in biomass allocation, including reproductive parts, along the drought gradients. Moreover, whether drought stress could alter the allometric trajectory of biomass between different organs still remains unclear, because previous studies about biomass allocation under drought stress mainly focused on proportional changes in biomass and gave little attention to allometric scaling of plants (e.g., Huang, Zhao, Zhou, Luo, & Mao, 2010; Huang et al., 2009). Even studies addressing the allometric aspects of biomass allocation remain contentious. For instance, Skarpaas et al. (2016) found that allometric scaling of leaf mass *vs*. total plant mass and flower mass *vs*. total plant mass of *Veronica alpine*,* Viola palustris,* and *Carex capillaris* changed with a rainfall gradient. In contrast, Wu, Shen, Zhang, and Shi (2013) found constant allometric patterns among 70 plant species along a precipitation gradient in the field of the Tibetan Plateau.

Herein, we conducted a meta‐analysis through synthesizing 164 published studies to explore the impacts of drought stress on biomass allocation among four organs (i.e., root, stem, leaf, and reproductive parts) from ratio‐based and allometric scaling perspectives. Specifically, we address the following four questions: (1) What are the overall impacts of drought stress on ratio‐based biomass allocation among different parts, especially including reproductive organs? (2) Are the patterns of ratio‐based biomass allocation in response to drought stress varied among different life forms? (3) Is there any plasticity in allometric scaling relationship between different structures of plants in response to drought stress? and (4) which theory (i.e., optimal partitioning theory or allocation partitioning theory) can better explain the patterns of biomass allocation?

## 2 MATERIALS AND METHODS

### 2.1 Data compilation

We searched published literatures from Web of Science (ISI), Google Scholar, Scopus, and China National Knowledge Infrastructure (CNKI) using key words “drought OR water stress OR rainout shelter” and “biomass allocation OR biomass partitioning OR biomass distribution.” We then screened the results based on the following criteria: (i) Papers should report any of the plant dry mass components (e.g., root, stem, leaf, and reproductive organs) and their ratios in total mass only under well‐watered (CK, hereafter) and controlled drought treatments; (ii) both drought and CK treatments should be started simultaneously under either a controlled (e.g., growth chamber, greenhouse, glasshouse, and nursery) or semicontrolled (rainout shelter) environment with the same soil type or substrate; (iii) plants under drought relative to plants under CK had to be water deficit in soil; and (iv) soil moisture under CK had to be lower than the field capacity of corresponding soil or substrate. According to these criteria, we compiled 1079, 682, 814, and 337 pairwise observations of root, stem, leaf, and aerial reproductive organ biomass, respectively, from 164 published papers (Appendix S1). For each publication, we recorded the experimental location, species name, life form, experimental settings (greenhouse, growth chamber, rainout shelter, etc.), treatment duration, and the response variables (biomass). For each observation, mean value, standard deviation (or standard error), and sample size were extracted directly from the tables or figures through Getdata Graph Digitizer 2.25 (http://www.getdata-graph-digitizer.com). If the standard deviation was absent, it was calculated through standard error multiplied by square root of sample size.

Plant life form is the result of the long‐term adaptation of plants to a given environment. Plants from the same life form generally have similar physiological traits and external morphology (Whittaker, 1977). In this study, we divided the plants into herbs and woody plants and then grouped the herbaceous plants into perennial and annual herbaceous plants. Moreover, we also classified the plants into cultivated and wild plants.

### 2.2 Statistical analysis

Reproductive mass in this study only included aerial parts such as flower, fruit, and seeds; rhizome was regarded as a part of the root mass. We calculated the biomass ratios based on the total mass without root mass or reproductive mass, as root mass or reproductive mass was not included in the total mass in some studies. Given that, our aim was to compare the difference in biomass allocation between plants in CK and drought treatments, the ratio‐based biomass allocation with or without some organs would not influence the results derived by the statistical analyses using effect size as follows. In some cases, root mass (RM) and root mass fractions (RMF) cannot be obtained directly from the tables or the figures in the published studies. In this case, we calculated them using Equation (1).

(1) $$\text{RM}=\frac{\text{RS}\times \text{TM}}{1+\text{RS}};\;\;\text{RMF}=\frac{\text{RS}}{1+\text{RS}}$$

where TM and RS are total mass and root mass to shoot mass ratio, respectively.

Natural log of the response ratio (Ln RR), namely effect size (Hedges, Gurevitch, & Curtis, 1999), was employed to analyze the overall effects of drought on biomass allocation. Response ratio (RR) was calculated as a ratio of biomass allocation value in the drought treatment (*x*_*d*_) to that in the CK treatment (*x*_*c*_) (Equation (2)). The log transformation is more favorable to conduct statistical analysis (Hedges et al., 1999).

(2) $$\text{Ln RR}=\text{Ln}\frac{\bar{x_{d}}}{\bar{x_{c}}}=\text{Ln}\;\bar{x_{d}}-\text{Ln}\;\bar{x_{c}}$$

In general, effect size Ln RR is considered to follow a normal distribution and was usually fitted with Gaussian curve (Bai et al., 2013). Variances of response ratio (*v*) were calculated using the following equation:

(3) $$v={\left({\text{SD}}_{d}\right)}^{2}/n_{d}\bar{x_{d}^{2}}+{\left({\text{SD}}_{c}\right)}^{2}/n_{c}\bar{x_{c}^{2}}$$

where SD_*d*_ and SD_*c*_ are the standard deviations of biomass allocation for drought and CK treatments, respectively; *n*_*d*_ and *n*_*c*_ are the sample sizes for the respective treatments. We calculated the SD for some studies using the mean value multiplied by the average coefficient of variance (CV) of each complete data set (He & Dijkstra, 2014).

The mean effect sizes were calculated by applying the random‐effects model in Meta Win 2.1 (Rosenberg, 2000), which is suitable for reflecting differences among experimental conditions and ecosystems (Wang et al., 2015). If the Ln RR < 0, effect is considered as negative and vice versa. The 95% confidence intervals (95% CI) were calculated by bootstrapping with 4999 iterations. Drought treatment was considered as statistically significant when the 95% CIs did not include zero. The mean effect sizes (Ln RR) were converted to antilog form (100*(RR‐1)), and effects of drought were reported as a proportional change compared with controls. To examine the possible biases of publication with multiple observations, we compared mean effect size of whole data set and the effect size calculated based on one random observation from each study (He & Dijkstra, 2014). No discrepancies were detected between these two approaches, indicating that over‐representation was less likely to occur in this study (Fig. S1). We used between‐group heterogeneity (*Q*_*between*_) to determine the differences in effect size between different life forms and tested the significance of *Q*_between_ based on the critical value in a standard chi‐square table (Zvereva, Lanta, & Kozlov, 2010).

We performed the reduced major axis (RMA) regression to investigate the scaling relationships between biomass in different plant organs. All data were log‐transformed before analyses. Log‐transformed linear equations were used to describe the allometric relationships between organs (Equation (4)).

(4) logy=α(logx)+β

where *y* and *x* represent the biomass of two different organs, α is the scaling exponent, and β is the normalization constant (Niklas, 1993; Weiner, 1990; West et al., 1997). We conducted a likelihood ratio test to detect the heterogeneity of RMA regression exponents between drought and CK treatments (Warton, Wright, Falster, & Westoby, 2006). We also performed general linear model (GLM) to further investigate the effects of drought and plant body size on biomass allocation. All statistical analyses were performed using R 3.2.3 (R Core Team, 2015).

## 3 RESULTS

### 3.1 Drought effect on biomass allocation

**Figure 1:**
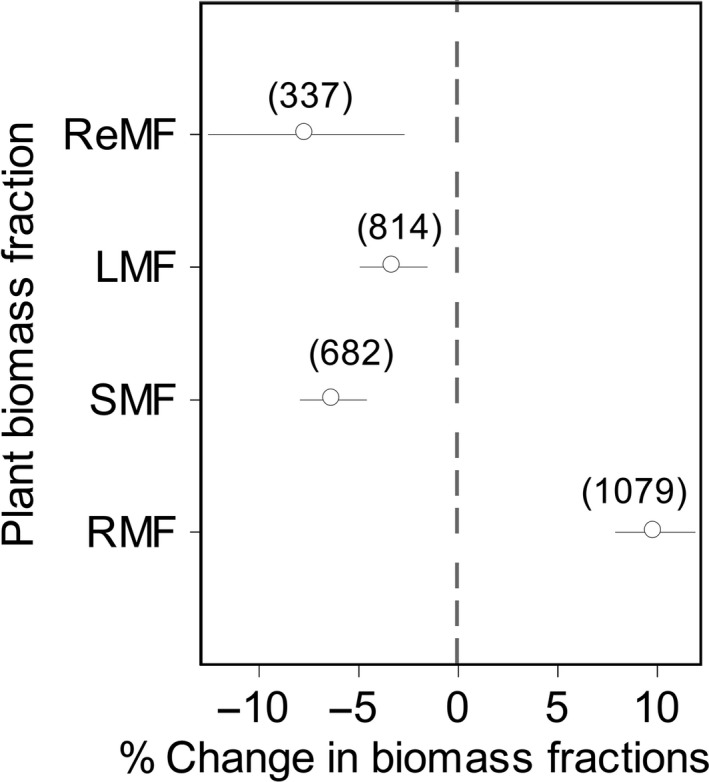
Effect of drought on fraction of root, stem, leaf, and reproductive biomass. Error bars show the 95% confidence intervals (CIs). Numbers of observations were given in the brackets. The effects of drought are expressed as percentage change relative to the control (%)

On average, drought increased root mass fraction (RMF) by 9.07% (95% CI = 6.93–10.40%) but decreased stem mass fraction (SMF), leaf mass fraction (LMF), and reproductive mass fraction (ReMF) by 5.55% (95% CI = 4.04–7.50%), 2.29% (95% CI = 0.35–3.62%), and 7.54% (95% CI = 3.36–12.51%), respectively (Figure 1).

**Figure 2:**
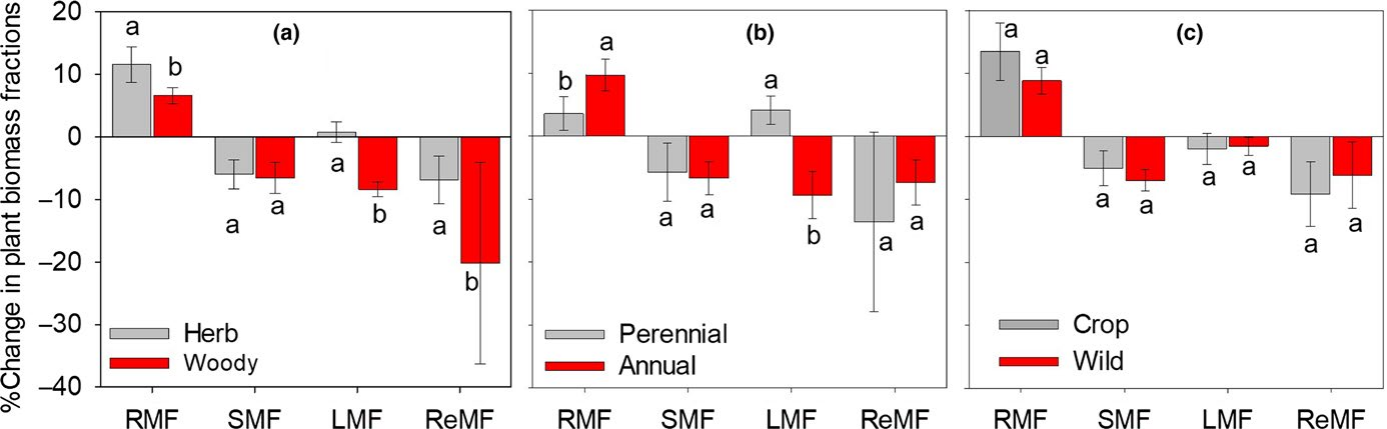
Effect of drought on biomass fraction of root (RMF), stem (SMF), leaf (LMF), and reproductive (ReMF) in different plant forms. (a) herbaceous and woody plants, (b) perennial and annual herbaceous plants, and (c) cultivated and wild plants. Error bars show the 95% confidence intervals (CIs). Different letters indicate significant difference of the response ratios based on heterogeneity test. Effects of drought are expressed as percentage change relative to the control (%)

Drought effects on biomass allocation ratios differed across life forms. Drought had a more positive impact on the RMF of herbs than that of woody plants (11.58% *vs*. 6.64%; *Q*_between_ = 4.91, *p *<* *.05) (Figure 2a & Table S1). In contrast, drought decreased the SMF of both woody and herbaceous plants similarly (5.97% *vs*. 6.55%; *Q*_between_ = 0.11, *p *=* *.74). Drought caused more decrease in the LMF of woody plants than in herbaceous plants (−8.38% *vs*. 0.79%; *Q*_between_ = 26.10, *p *<* *.01). (Figure 2a & Table S1). Drought had a more negative impact on the ReMF of woody plants than that of herbaceous plants (20.19% *vs*. 8.15%; *Q*_between_ = 8.31, *p *<* *.05) (Figure 2a & Table S1).

In comparison with annual herbs, biomass allocation of perennial herbs showed different responses to drought. Drought had similar impacts on the SMF and ReMF for the two life forms but increased RMF of annual herbs more than that of perennial herbs (9.70% *vs*. 5.44%; *Q*_between_ = 4.14, *p *<* *.05). Remarkably, drought had a significant negative effect on the LMF for annual herbs (average decrease of 9.38%), whereas the LMF for perennial herbs increased slightly by 4.13% on average in response to drought (Figure 2b). In addition, drought showed no contrasting influences on biomass allocation (*i.e*., RMF, SMF, LMF, and ReMF) of wild and cultivated plants (Figure 2c).

### 3.2 Drought effects on allometric relationships between biomass of different organs

**Figure 3:**
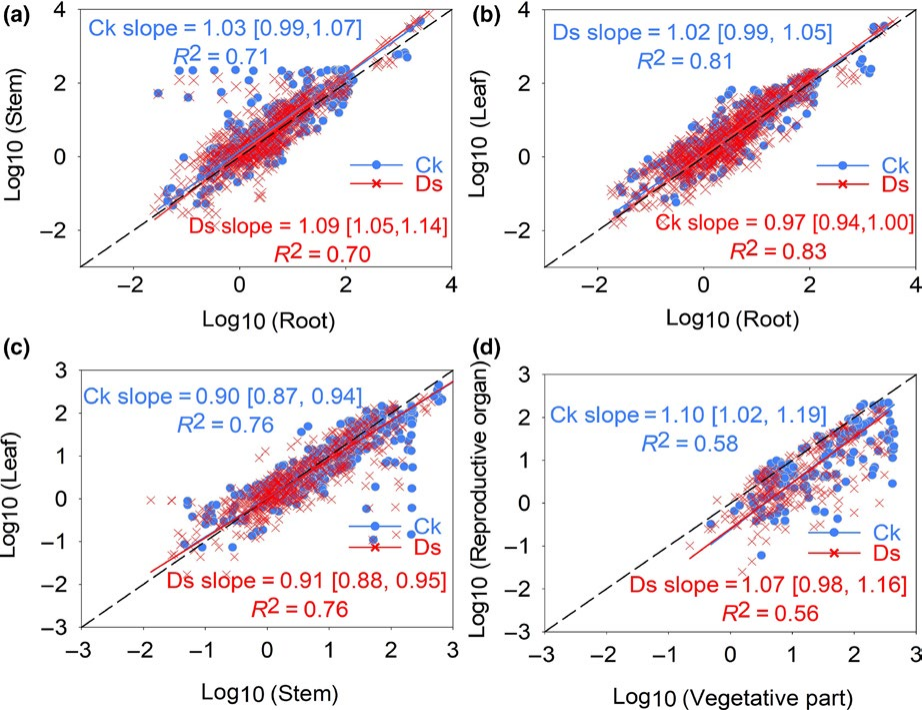
Allometric relationships among different plant organs. (a) stem vs. root mass; (b) leaf vs. root mass; (c) leaf vs. stem mass; and (d) reproductive vs. vegetative mass. RMA regression was used to determine the significant line (*p *<* *.05). Numbers in square brackets are the lower and upper 95% confident intervals of the RMA slopes. All data are log_10_‐transformed before analysis

Scaling exponents of stem *vs*. root, leaf *vs*. root, leaf *vs*. stem, and reproductive *vs*. vegetative (which consists of root, stem, and leaf) biomass were 1.03, 1.02, 0.90, and 1.10 under CK treatment, respectively, and were 1.09, 0.97, 0.91, and 1.07 under drought treatment, respectively (Figure 3 and Table S2). These revealed that there were no significant differences in the allometric scaling exponents between the drought and CK treatments (Figure 3 & Table S3), supporting the allometric partitioning theory. For same species, we also found no significant differences in the scaling exponents between the drought and CK treatments (Fig. S3). GLM analysis showed that plant size explained 5.81%, 2.57%, 5.27%, and 0.65% of variation in allocation ratios for RMF, SMF, LMF, and ReMF, respectively, which were much higher than that of the treatment (Table 1), indicating that the plant size had stronger impact on biomass allocation ratios.

**Table 1:** Summary of the general linear model for biomass fractions of root (RMF), stem (SMF), leaf (LMF), and reproductive (ReMF) in all plants under control and drought treatments

Traits	Items	*df*	MS	F	*p*	SS%
RMF	Treatment	1	0.35	12.16	.00	0.53
	Plant size	1	3.79	132.11	.00	5.81
	Residuals	2131	0.03			93.66
SMF	Treatment	1	0.09	3.96	.05	0.29
	Plant size	1	0.82	34.59	.00	2.57
	Residuals	1307	0.02			97.14
LMF	Treatment	1	0.02	0.56	.46	0.03
	Plant size	1	2.85	86.83	.00	5.27
	Residuals	1559	0.03			94.69
ReMF	Treatment	1	0.06	1.58	.21	0.24
	Plant size	1	0.17	4.32	.04	0.65
	Residuals	663	0.04			99.12

DF, degrees of freedom; MS, mean squares; SS, proportion of variances explained by the variable.

## 4 DISCUSSION

### 4.1 Drought effect on biomass allocation from the ratio‐based perspective

Our analyses showed that plants tend to increase their root system investments at the expense of shoot mass with drought (Figure 1). This is consistent with a previous study that found higher RMF and lower LMF and SMF under water scarce condition (Poorter et al., 2012). With a relatively proliferated root system, plants are better able to tap water from deep soil. The stem transports water and nutrients from the root to other parts of the plant and provides architectural support for leaves and reproductive organs (Stock, Heyden, & Lewis, 1992). The leaf, as another major outlet of water via transpiration, is a pivotal component of photosynthesis (Lambers et al., 2008). Under drought, plants would invest less to the stem and leaf to reduce the water loss to minimum level. Thus, SMF and LMF reduce significantly (Farooq, Hussain, Wahid, & Siddique, 2012; Mendez & Karlsson, 2007; Pereira & Chaves, 1995). Similarly, plants also tended to reduce the reproductive investment (Figure 1) that requires vast amounts of energy and water (Karlsson & Méndez, 2005), because reduction in photosynthesis (Su et al., 2013) and changes in phenology (e.g., shortening growth period and early flowering) in relation to drought cause significant reduction in biomass allocation to reproductive parts (Farooq, Wahid, Kobayashi, Fujita, & Basra, 2009; Farooq et al., 2012).

The drought‐induced responses of biomass allocation differed between herbs and woody plants, which may be attributed to the distinct ontogenetic ways and physiological structures, such as plant size and life spans (Breshears & Barnes, 1999). Compared with herbs, woody plants are relatively bigger in size, and have more investment of resources in supportive structures, and grow relatively slower (Stock et al., 1992). These differences may cause their different responses of biomass allocation to drought (Chiatante, Di Iorio, Maiuro, & Scippa, 1999). Our results showed that under drought, indeed, the RMF of herbaceous plants developed more rapidly than woody plants did (Figure 2a). The relatively big and deep root system in woody plants can buffer the effects of drought, while herbaceous plants with their small proportional and shallow root systems tend to be affected more strongly (Chiatante et al., 1999; Nepstad et al., 1994). Consistently, one previous study from a field experiment found that the RMF of shallow‐rooted herbaceous plants increased much faster than deep‐rooted shrubs (West et al., 2012). However, drought induced greater decrease in the ReMF of woody plants than that of herbs (Figure 2a). Similarly, Zhao et al. (2012) also found a significant reduction in reproductive allocation of *Lycium barbarum* (shrub) under drought, while Mahieu et al. (2009) reported relatively minor changes in reproduction of *Pisum sativum* (annual herb). Current reproduction may be relatively less important for woody than herbaceous plants (Silvertown, Franco, Pisantybaruch, & Mendoza, 1993). Thus, even when resources are scarce, vegetative parts of woody plants remain relatively stable at the expense of reproductive growth (Delerue, Gonzalez, Atlan, Pellerin, & Augusto, 2013; Li, Peng, Chen, & Hou, 2010). Additionally, we also found that drought had more negative impact on the LMF of woody than herbaceous plants (Figure 2a). A possible explanation could be that relative to herbaceous plants, woody plants tend to close their stomata earlier to prevent xylem cavitation (Vilagrosa, Bellot, Vallejo, & Gil‐Pelegrin, 2003) under drought. This may depress the photosynthesis of woody plants more than that of herbaceous plants, which presumably result in more reduction in LMF of woody plants (Camachob, Hall, & Kaufmann, 1974). In contrast, our analysis suggested that effects of drought on the SMF were similar for both woody and herbaceous plants (Figure 2a), presumably because most of the woody plants in this study consist of young seedlings that are in some ways anatomically “herbaceous” (Chiatante et al., 1999).

Among the herbaceous plants, the RMF of perennial herbs was less sensitive to drought stress than that of annuals (Figure 2b) because annual herbs with relatively short life spans are the typical example of stress‐avoiding strategies of plants (Pitelka, 1977). They invest more in current reproduction and growth but less in storage and defense mechanisms such as roots and stems (Pitelka, 1977). By contrast, perennial herbs that are relatively bigger in size and resource‐conserving species invest more in defense and storage (Roumet, Urcelay, & Diaz, 2006). Thus, annual herbs are more likely to be affected by abiotic stresses (Chapin, Autumn, & Pugnaire, 1993; Roumet et al., 2006). Interestingly, with drought, the LMF for perennial herbs showed mild increase while that for annuals reduced significantly, possibly due to the ontogenetic difference of plant size (Poorter et al., 2012).

Drought showed no contrasting effects on biomass allocation ratios for cultivated and wild plants (Figure 2c). In prior studies, Schulze et al. (1980) found no consistent trend of drought tolerance among cultivated and wild plants, while Nevo and Chen (2010) found a greater drought resistance in wild wheat and barley than cultivated counterparts. By contrast, some others suggested that drought tolerant‐targeted transgenetic cultivated plants had strong resistance to drought than even wild plants (Fita, Rodriguezburruezo, Boscaiu, Prohens, & Vicente, 2015; Seversike, 2011; 1998,b; Watanabe, Kikuchi, Shimazaki, & Asahina, 2011). Taken together, these comparisons suggested that drought tolerance of crops and wild plants may be species‐specific.

### 4.2 Drought effects on biomass allocation from the perspective of allometric relationships

Plant allometry is a way to interpret plant biomass allocation to different functions and structures as a function of plant size (Müller et al., 2000). In other words, biomass ratios of plant structures will change with plant size (Weiner, 2004). In a certain period of environmental constraint, plants tend to maintain a specific allometric trajectory, because it is always desirable to have an evolved simple allometric strategy than complex various trajectory according to the availability of particular resources. Given that plants are small in resource‐poor but large in resource‐rich environments, a single trajectory can meet the needs of the plants with optimal biomass ratios of different organs (Müller et al., 2000).

In our study, we found that the RMF and LMF reduced, while the SMF increased with plant size (Fig. S2). The GLM analysis provided an evidence that plant size has a significant impact on allocation ratios (Table 1), because when a plant is small (or water is scarce), root mass proportion is relatively large, but it is shifted to the leaf and eventually to the stem with increasing plant size (Tilman, 1989). A previous study also supported that biomass allocation is size‐dependent (Müller et al., 2000). Even though drought causes significant changes in the absolute value of plant biomass and allocation ratios, as allometric theory predicts, the scaling relationship between the biomass of organ pairs remained stable (Figure 3). This result may be attributed to the fact that the proportional relationship between relative growth rates of different organs is quite stable throughout the organism's life history, irrespective of short‐term environmental fluctuation (Huxley, 1924; Huxley, 1932; Niklas, 1994). In addition, although sharp differences existed in allometric relationships of different organ pairs among different species and life forms, the effects of drought on the allometric relationships are rarely effective (Figs S3 & S4). Cheng, Wang, Tang, Li, and Zhong (2009) also reported similar results that along a soil moisture gradient, the allometric relationship between the root mass and shoot mass of different plants was stable. Wu et al. (2013) reported that the allometric relationship between vegetative structures of 70 individual species on the Tibetan Plateau did not change with a rainfall gradient, but it differed from each other in allometric relationships of different organs across the life forms. These results indicated that the patterns of ratio‐based biomass allocation might be induced by changes in plant size along the allometric trajectory. Notably, much of the variances of ratio‐based biomass allocation could not be explained by the treatment and plant size (Table 1), which may be attributed to the differences in ontogenetic processes, plant individuals, species, and other environmental factors.

## 5 CONCLUSION

Our results showed that the SMF, LMF, and ReMF significantly decreased while the RMF increased in response to drought stress. The RMF of woody plants was less sensitive to drought compared to herbaceous plants that reduced the ReMF less than woody plants. Meanwhile, drought had a more negative effect on the LMF of woody plants than herbaceous plants. The RMF of annual herbs was more plastic than that of perennial herbs that reduced ReMF more than annuals did in response to drought. Both cultivated and wild plants responded to drought stress in similar ways. These findings highlighted that extreme climatic events such as drought may shift the plant community structure due to unequal effects of drought among different life forms (Baez, Collins, Pockman, Johnson, & Small, 2013; Churchillamber, Turetskymerritt, David, & Hollingsworthteresa, 2015). Although the biomass proportion of plant structures changed with drought stress, drought had almost no impact on allometric relationship among different parts of plants, indicating that plant biomass allocation patterns in relation to drought stress were better explained by the allometric partitioning theory.

## CONFLICT OF INTEREST

None declared.

## AUTHORSHIP

Anwar Eziz, Jingyun Fang, and Zhengbing Yan designed the research. Anwar Eziz performed the research and analyzed the data, and Anwar Eziz, Zhengbing Yan, Wenxuan Han, Zhiyao Tang, Di Tian, and Jingyun Fang wrote the manuscript.
