# Supplementary figures and images for: Drought effect on plant biomass allocation: A meta‐analysis
Source: Ecol Evol. 2017 Nov 12;7(24):11002–10. doi: 10.1002/ece3.3630 (PMC5743700; doi:10.1002/ece3.3630)

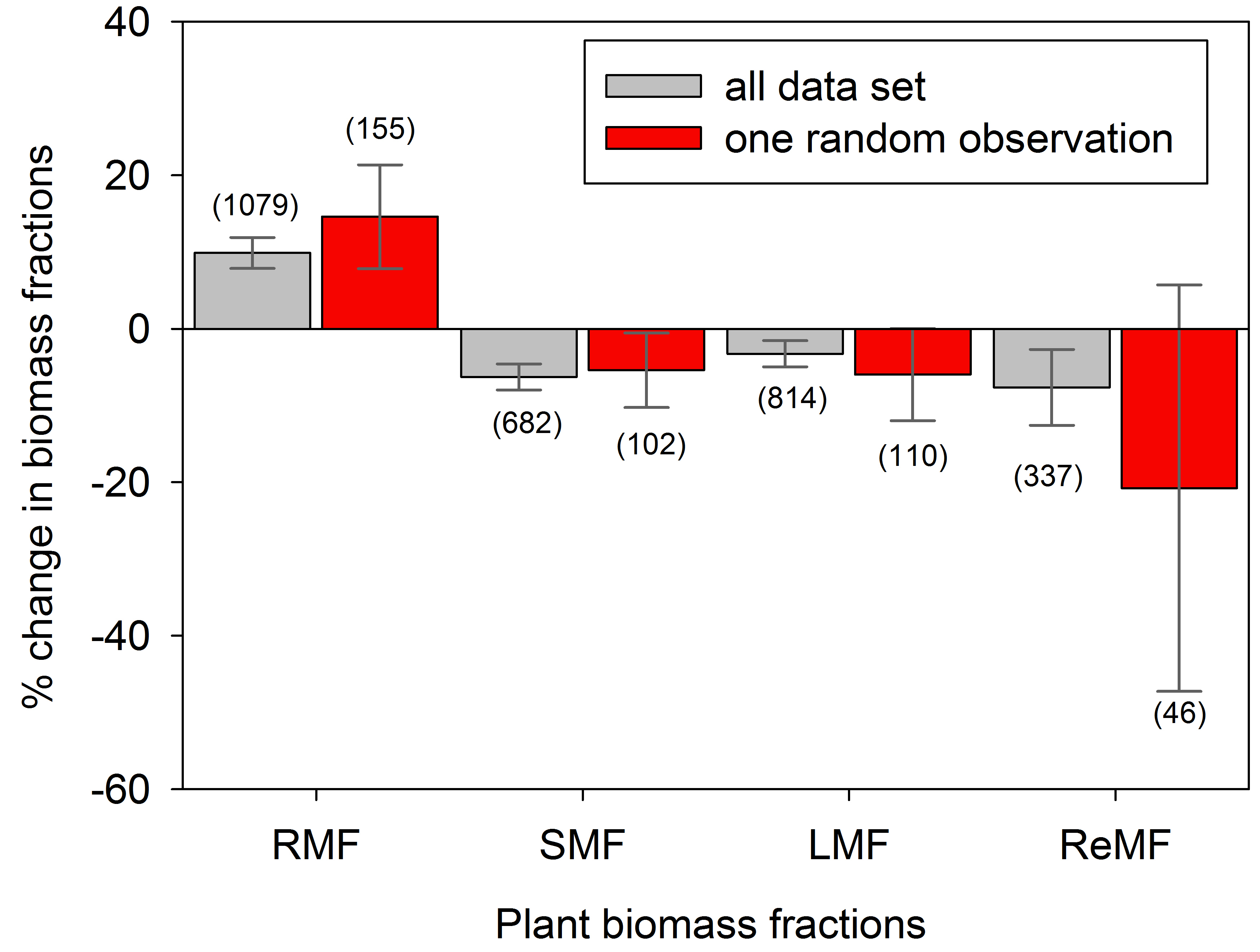

Supplement: Supplementary file 1 [file ECE3-7-11002-s001.tif]

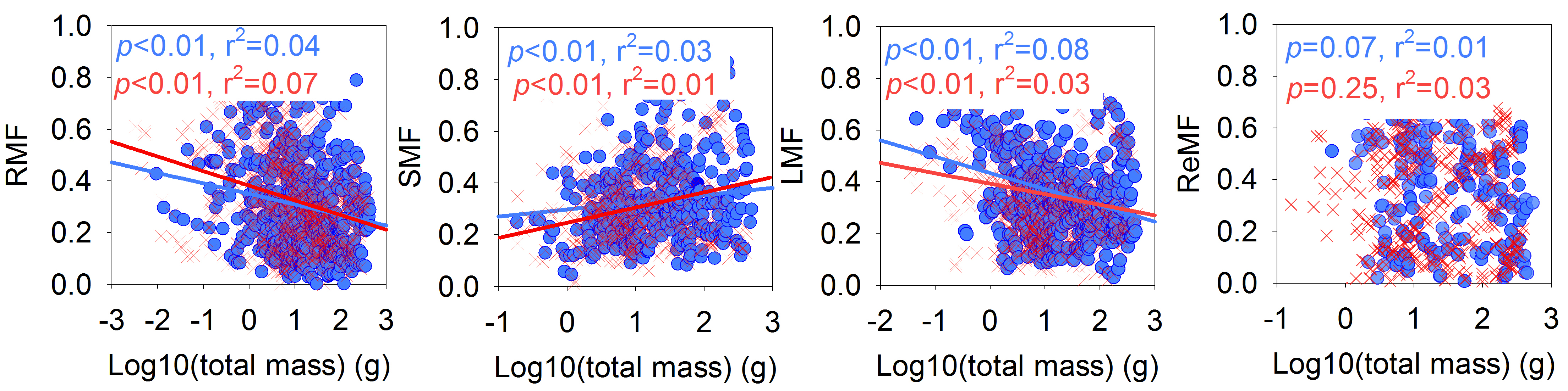

Supplement: Supplementary file 2 [file ECE3-7-11002-s002.tif]

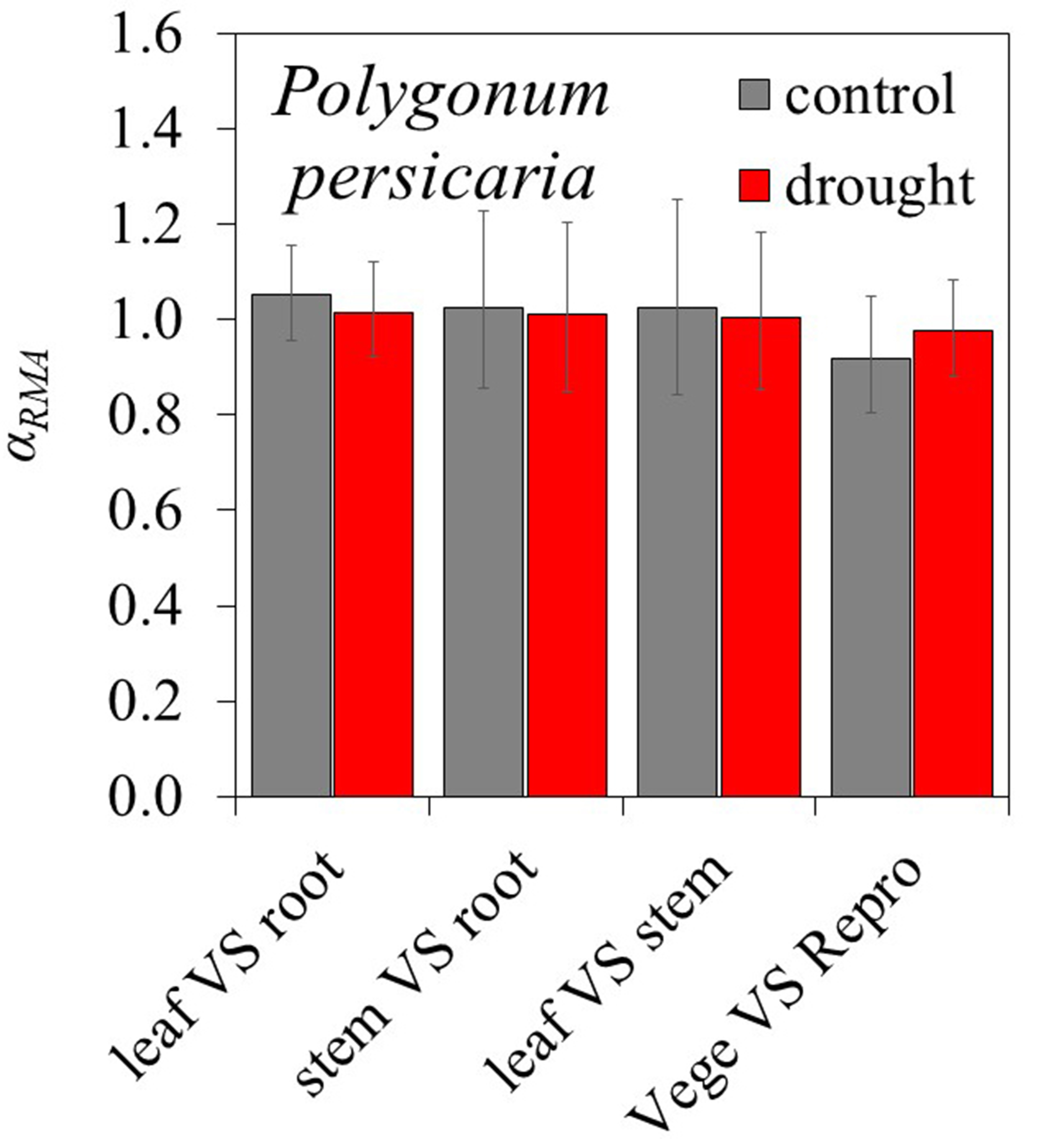

Supplement: Supplementary file 3 [file ECE3-7-11002-s003.tif]

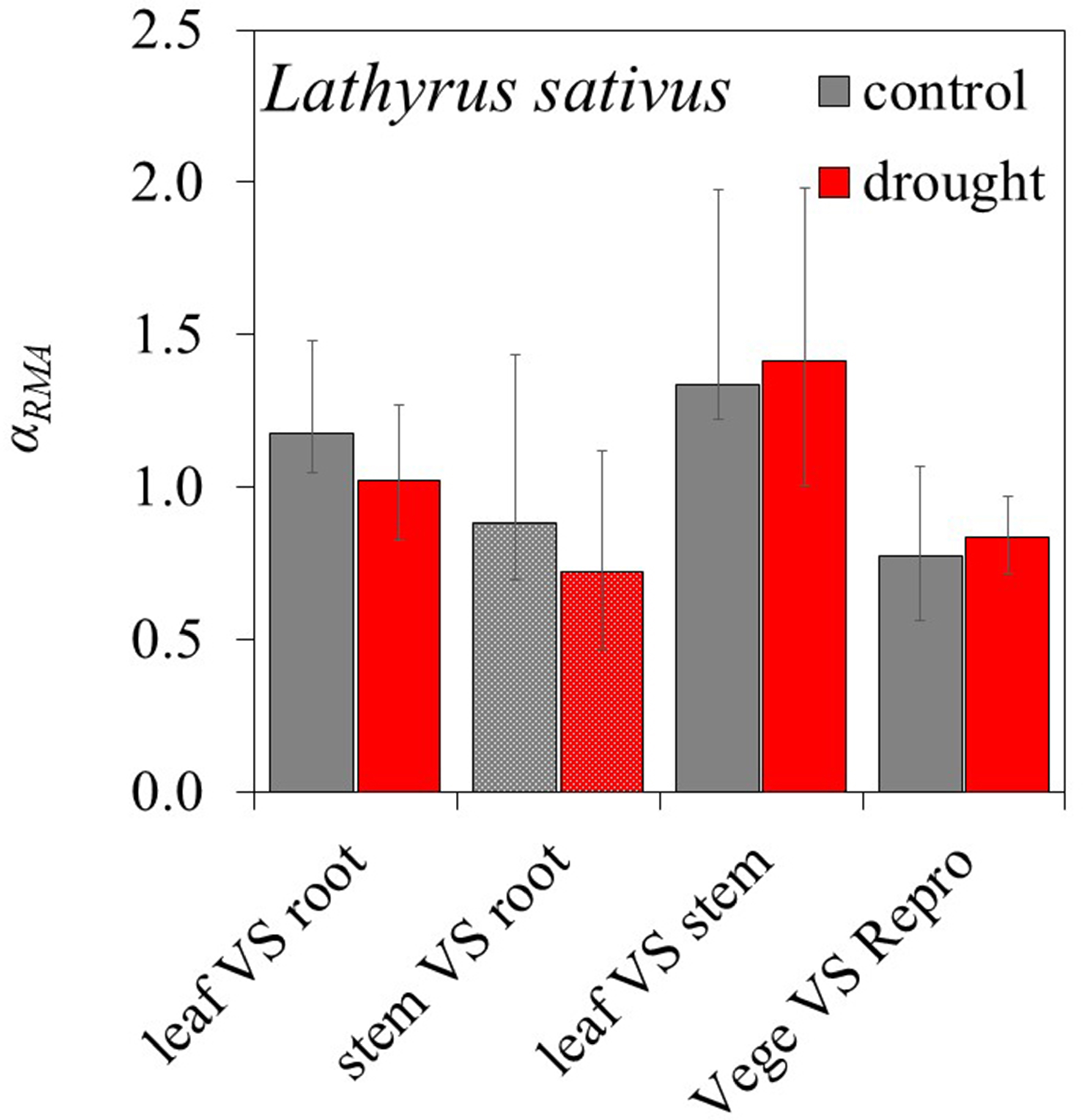

Supplement: Supplementary file 4 [file ECE3-7-11002-s004.tif]

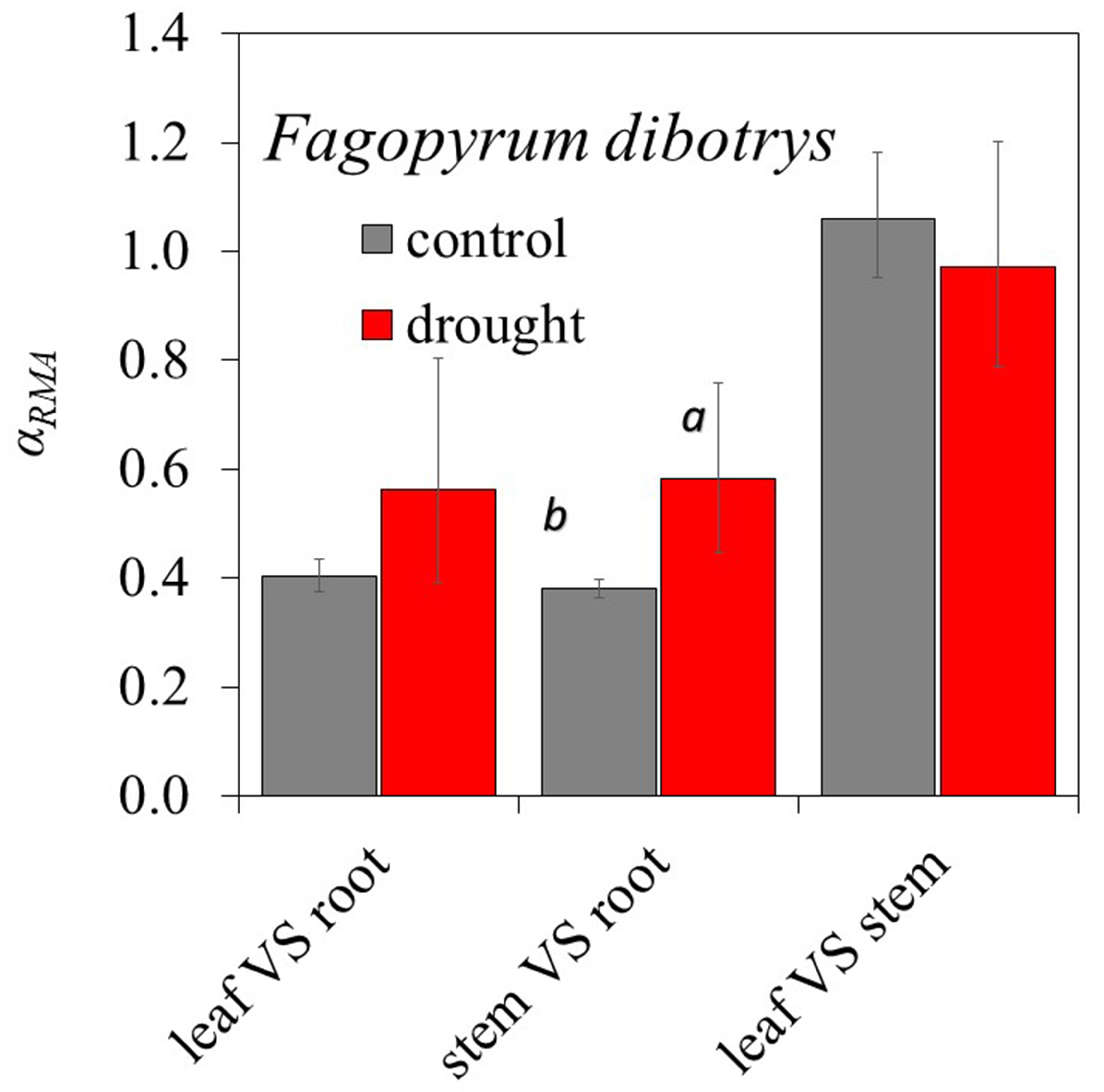

Supplement: Supplementary file 5 [file ECE3-7-11002-s005.tif]

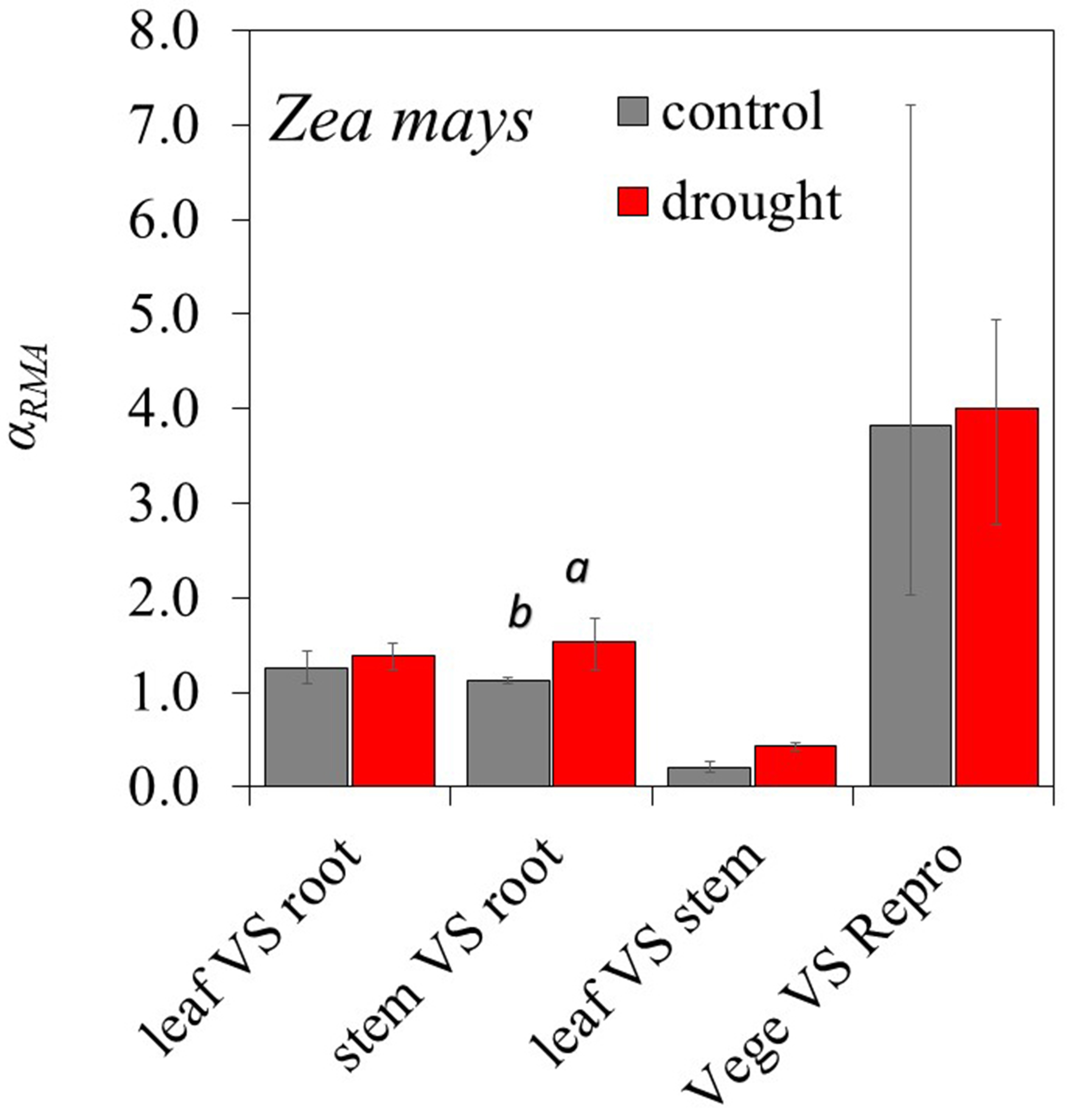

Supplement: Supplementary file 6 [file ECE3-7-11002-s006.tif]

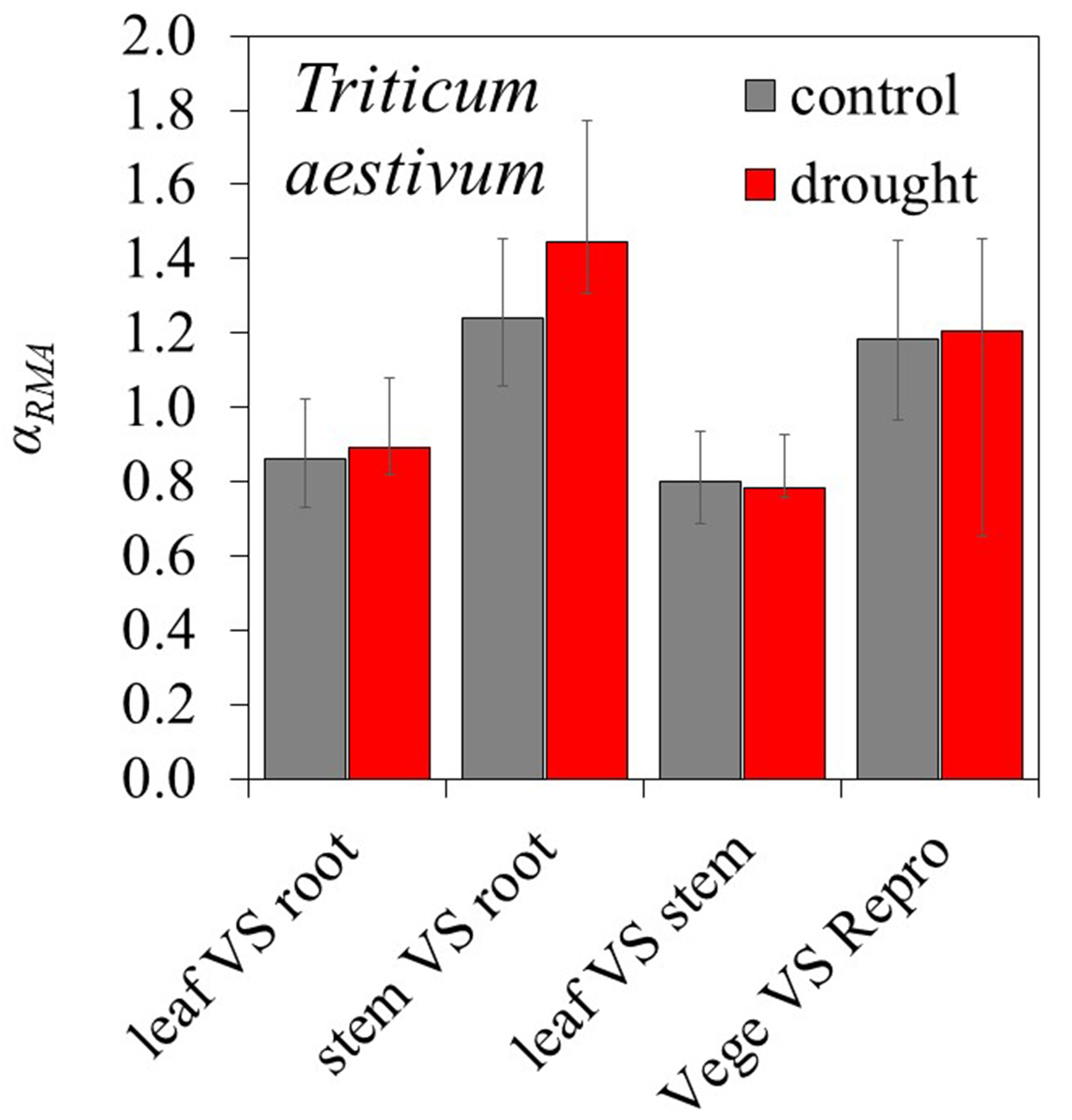

Supplement: Supplementary file 7 [file ECE3-7-11002-s007.tif]

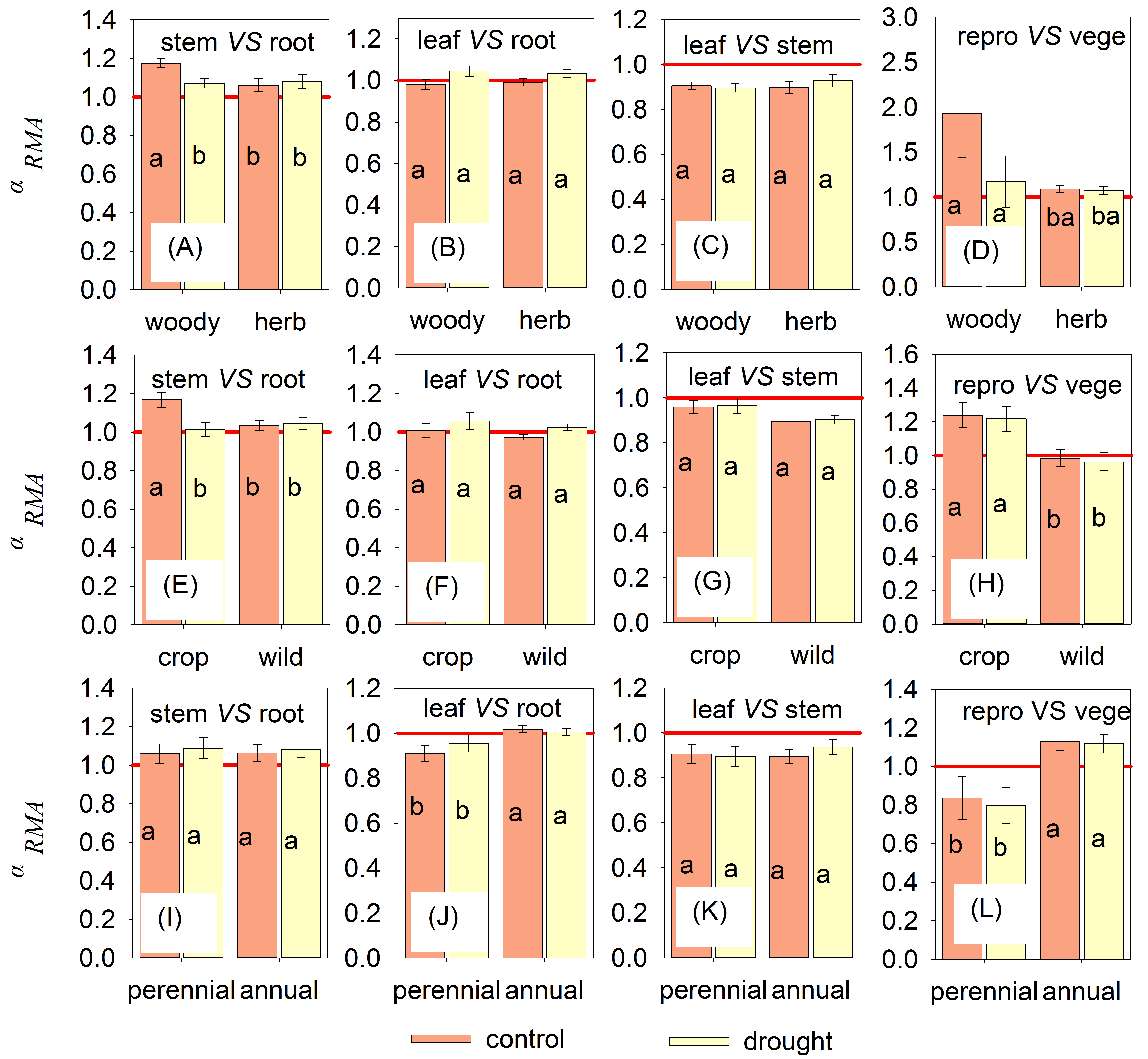

Supplement: Supplementary file 8 [file ECE3-7-11002-s008.tif]
