# Supplementary material for: Drought effect on plant biomass allocation: A meta‐analysis
Source: Ecol Evol. 2017 Nov 12;7(24):11002–10. doi: 10.1002/ece3.3630 (PMC5743700; doi:10.1002/ece3.3630)
Supplement: Supplementary file 9 [file ECE3-7-11002-s009.docx]

**SUPPORTING INFORMATION**

**Drought effect on plant biomass allocation: a meta-analysis**

Anwar Eziz^1^*, Zhengbing Yan^1^, Di Tian^1^, Wenxuan Han^2^, Zhiyao Tang^1^, Jingyun Fang^1^

*1* *Department of Ecology, College of Urban and Environmental Sciences, Peking University, Beijing 100871, China*

*2 Key Laboratory of Plant-Soil Interactions, Ministry of Education, College of Resources and Environmental Sciences, China Agricultural University, Beijing 100193, China*

**Corresponding author Anwar Eziz; Email:* [*anwareziz@pku.edu.cn*](mailto:anwareziz@pku.edu.cn)

**This file includes:**

**Figures S1-S3**

**Tables S1**–**S4**

**Appendix 1**

**Supplementary Figures S1-S4**

**Figure S1** Effect of drought on fraction of root mass (RMF), stem mass (SMF), leaf mass fraction (LMF) and reproductive mass (ReMF) calculated by using the complete dataset and by using one random observation per study. Error bars show the 95% confidence intervals (CIs). Numbers of observations were given in the brackets. Different letters indicate significant difference in the response ratios based on heterogeneity test. The effects of drought are expressed as percentage change relative to the control (%).

**Figure S2** Relationship between ratio-based biomass allocation and total biomass under control (light blue circle) and drought stress (red cross). RMF, root mass fraction; SMF, stem mass fraction; LMF, leaf mass fraction; ReMF, reproductive mass fraction.

**Figure S3** The RMA regression slopes of biomasses across pairwise organ combinations in *Zea mays*, *Triticum aestivum*, *Lathyrus sativus*, *Polygonum persicaria* and *Fagopyrum dibotrys* under drought and control conditions. Different letters indicate significant difference (*p* < 0.05) based on a maximum likelihood ratio test. All the slopes are significant except for *stem VS. root* in *Lathyrus sativus* (*p*>0.05). Error bars show the slope and the 95% confidence interval (CI).

**Figure S4** Comparisons of the RMA regression slopes (*α_RMA_*) of the allometric relationships under drought stress (Ds) and well watered (Ck) condition among woody, herbaceous, annuals, perennial herbs, crops and wild plants. Error bars show the slope and the 95% confidence interval (CI). Different letters indicate significant difference (*p* < 0.05) based on a maximum likelihood ratio test.. The words ‘repro’ and ‘vege’ are the abbreviation forms of reproductive mass and vegetative mass, respectively.

# Supplementary Tables S1-S3

**Table S1** Effect of drought on fraction of root mass (RMF), stem mass (SMF), leaf mass (LMF) and reproductive mass (ReMF). Mean effects of drought was expressed as percentage change relative to the control (%) and was calculated through aniloging the response ratio (Exp (Ln*RR*)-1)*100. and the 95% confidence intervals (CIs) are provided here. Differences in the effects of drought among different life forms are indicated by *Q_between_* and *P_random_* based on heterogenity test.

| Plant Biomass | | Category | Groups | n | (*RR*-1)*100 (%) | 95% CI | | Q_between_ | | *p*_random_ |
| --- | --- | --- | --- | --- | --- | --- | --- | --- | --- | --- |
|  |  | |  |  |  | min | max |  | |  |
| RMF | Life forms | | Woody | 476 | 6.64 | 5.35 | 7.93 | 4.91 | | **0.04** |
|  |  |  | Herb | 603 | 11.58 | 8.71 | 14.46 |  | |  |
|  |  |  | Crop | 204 | 13.47 | 8.93 | 18.02 | 1.61 | | 0.25 |
|  |  |  | Wild | 875 | 8.84 | 6.78 | 10.90 |  | |  |
|  |  |  | Perennial | 211 | 3.57 | 0.90 | 6.24 | 4.14 | | **0.04** |
|  |  |  | Annual | 392 | 9.70 | 7.22 | 12.19 |  | |  |
| SMF | Life forms | | Woody | 309 | -6.55 | -9.04 | -4.05 | 0.11 | | 0.74 |
|  |  |  | Herb | 373 | -5.97 | -8.33 | -3.62 |  | |  |
|  |  |  | Crop | 186 | -5.71 | -10.29 | -1.13 | 0.09 | | 0.75 |
|  |  |  | Wild | 496 | -6.65 | -9.26 | -4.03 | |  |  |
|  |  |  | Perennial | 401 | -5.83 | -10.73 | -1.35 | | 0.00 | 1.00 |
|  |  |  | Annual | 284 | -5.82 | -8.16 | -3.33 | |  |  |
| LMF | Life forms | | Woody | 292 | -8.38 | -10.78 | -6.12 | | 26.10 | **0.00** |
|  |  |  | Herb | 522 | 0.79 | -1.61 | 3.01 | |  |  |
|  |  |  | Crop | 189 | -1.97 | -4.44 | 0.50 | | 0.01 | 0.94 |
|  |  |  | Wild | 625 | -1.55 | -2.97 | -0.13 | |  |  |
|  |  |  | Perennial | 159 | 4.13 | 1.87 | 6.40 | | 41.34 | **0.00** |
|  |  |  | Annual | 362 | -9.38 | -13.15 | -5.61 | |  |  |
| ReMF | Life forms | | Woody | 19 | -20.19 | -36.30 | -4.09 | | 8.31 | **0.02** |
|  |  |  | Herb | 318 | -6.85 | -10.68 | -3.02 | |  |  |
|  |  |  | Crop | 152 | -9.15 | -14.30 | -4.01 | | 0.62 | 0.55 |
|  |  |  | Wild | 185 | -6.13 | -11.42 | -0.84 | |  |  |
|  |  |  | Perennial | 27 | -13.65 | -27.91 | 0.61 | | 0.73 | 0.48 |
|  |  |  | Annual | 291 | -7.33 | -10.92 | -3.75 | |  |  |

**Table S2** Reduced major axis regression (RMA) results of biomasses among woody, herbaceous, annuals, perennial herbs, crops and wild plants under drought stress (Ds) and well watered (Ck) conditions. *α_RMA_* and *β_RMA_* are the regression slopes and constants. All data are log_10_-transformed before analysis. Different letters indicate significant difference (*p* < 0.05) based on a maximum likelihood ratio test.

| treatment | Pair groups | *β_RMA_* | *α_RMA_* | *β(95% CI)* | *α(95% CI)* | *r^2^* | n | *p* |
| --- | --- | --- | --- | --- | --- | --- | --- | --- |
| control | stem vs root | **0.15a** | 1.03a | (0.10~0.20) | (0.99~1.07) | 0.71 | 676 | 0.00 |
| drought | stem vs root | **0.04b** | 1.09a | (0.00~0.09) | (1.05~1.14) | 0.70 | 676 | 0.00 |
|  |  |  |  |  |  |  |  |  |
| control | leaf vs root | **0.06a** | 1.02a | (0.03~0.09) | (0.99~1.05) | 0.81 | 803 | 0.00 |
| drought | leaf vs root | **0.13b** | 0.97a | (0.10~0.16) | (0.94~1.00) | 0.83 | 803 | 0.00 |
|  |  |  |  |  |  |  |  |  |
| control | leaf vs stem | 0.01a | 0.90a | (0.03~0.05) | (0.87~0.94) | 0.76 | 666 | 0.00 |
| drought | leaf vs stem | 0.00a | 0.91a | (0.03~0.04) | (0.88~0.95) | 0.76 | 666 | 0.00 |
|  |  |  |  |  |  |  |  |  |
| control | repro vs vege | -0.62a | 1.10a | (-0.75~-0.49) | (1.02~1.19) | 0.58 | 264 | 0.00 |
| drought | repro vs vege | -0.59a | 1.07a | (-0.71~-0.47) | (0.98~1.16) | 0.56 | 264 | 0.00 |

**Table S3** Summary of the reduced major axis regression (*RMA*) results of root, stem, leaf, aerial reproductive part (repro), shoot and vegetative (vege) biomass of woody plants, herbs, crops, wild plants, perennial herbs and annual herbs under drought stress and control conditions. *α_RMA_* and *β_RMA_* are log-log transformed regression slopes and constants. The significance of the slopes was determined by using maximum likelihood ratio (the slopes with bold fonts are significantly different from one when p<0.05).

| Treatment | Pair groups | *β_RMA_* | *α_RMA_* | *β(CI 95%)* | *α(CI 95%)* | *R^2^* | *p* | *n* | *Notes* |
| --- | --- | --- | --- | --- | --- | --- | --- | --- | --- |
| drought | stem vs root | **-0.07c** | **1.07b** | (-0.12~-0.01) | (1.03~1.12) | 0.87 | 0.00 | 231 | woody |
| control | stem vs root | **-0.21b** | **1.17a** | (-0.26~-0.15) | (1.13~1.22) | 0.87 | 0.00 | 231 | woody |
| drought | stem vs root | 0.20a | 1.08b | (0.15~0.26) | (1.01~1.15) | 0.60 | 0.00 | 375 | herb |
| control | stem vs root | 0.28a | 1.06b | (0.21~0.34) | (1.00~1.13) | 0.61 | 0.00 | 375 | herb |
|  |  |  |  |  |  |  |  |  |  |
| drought | stem vs root | **0.19a** | **1.01b** | (0.12~0.26) | (0.94~1.09) | 0.67 | 0.00 | 234 | crop |
| control | stem vs root | **-0.02c** | **1.17a** | (-0.09~0.05) | (1.10~1.24) | 0.77 | 0.00 | 234 | crop |
| drought | stem vs root | 0.08ba | 1.05b | (0.03~0.14) | (0.99~1.11) | 0.65 | 0.00 | 413 | wild |
| control | stem vs root | 0.13a | 1.03b | (0.07~0.19) | (0.98~1.09) | 0.72 | 0.00 | 413 | wild |
|  |  |  |  |  |  |  |  |  |  |
| drought | stem vs root | 0.06b | 1.09a | (-0.03~0.16) | (0.99~1.20) | 0.75 | 0.00 | 102 | perennial |
| control | stem vs root | 0.17b | 1.06a | (0.08~0.26) | (0.97~1.16) | 0.78 | 0.00 | 102 | perennial |
| drought | stem vs root | 0.26a | 1.08a | (0.18~0.33) | (1.00~1.17) | 0.56 | 0.00 | 273 | annual |
| control | stem vs root | 0.32a | 1.06a | (0.24~0.40) | (0.98~1.15) | 0.56 | 0.00 | 273 | annual |
|  |  |  |  |  |  |  |  |  |  |
| drought | leaf vs root | **-0.14c** | 1.05a | (-0.19~-0.09) | (1.00~1.10) | 0.85 | 0.00 | 276 | woody |
| control | leaf vs root | **-0.01b** | 0.98a | (-0.07~0.04) | (0.93~1.03) | 0.84 | 0.00 | 276 | woody |
| drought | leaf vs root | 0.15a | 1.03a | (0.12~0.19) | (1.00~1.07) | 0.82 | 0.00 | 527 | herb |
| control | leaf vs root | 0.19a | 0.99a | (0.15~0.22) | (0.96~1.03) | 0.83 | 0.00 | 527 | herb |
|  |  |  |  |  |  |  |  |  |  |
| drought | leaf vs root | 0.27a | 1.06a | (0.21~0.33) | (0.98~1.14) | 0.73 | 0.00 | 175 | crop |
| control | leaf vs root | 0.31a | 1.01a | (0.25~0.36) | (0.94~1.08) | 0.78 | 0.00 | 175 | crop |
| drought | leaf vs root | **0.00c** | 1.02a | (-0.04~0.03) | (0.99~1.06) | 0.84 | 0.00 | 628 | wild |
| control | leaf vs root | **0.07b** | 0.97a | (0.04~0.10) | (0.94~1.00) | 0.85 | 0.00 | 628 | wild |
|  |  |  |  |  |  |  |  |  |  |
| drought | leaf vs root | 0.31a | 0.95b | (0.28~0.35) | (0.92~0.99) | 0.88 | 0.00 | 359 | perennial |
| control | leaf vs root | 0.34a | 0.91b | (0.31~0.38) | (0.88~0.94) | 0.88 | 0.00 | 359 | perennial |
| drought | leaf vs root | -0.12b | 1.01a | (-0.18~-0.06) | (0.93~1.08) | 0.77 | 0.00 | 168 | annual |
| control | leaf vs root | -0.05b | 1.02a | (-0.11~0.01) | (0.95~1.09) | 0.80 | 0.00 | 168 | annual |
|  |  |  |  |  |  |  |  |  |  |
| drought | leaf vs stem | 0.02a | 0.90a | (0.02~0.06) | (0.86~0.93) | 0.89 | 0.00 | 277 | woody |
| control | leaf vs stem | 0.05a | 0.90a | (0.01~0.09) | (0.87~0.94) | 0.90 | 0.00 | 277 | woody |
| drought | leaf vs stem | -0.01b | 0.93a | (-0.07~0.04) | (0.87~0.98) | 0.65 | 0.00 | 389 | herb |
| control | leaf vs stem | -0.01b | 0.90a | (-0.07~0.05) | (0.85~0.95) | 0.66 | 0.00 | 389 | herb |
|  |  |  |  |  |  |  |  |  |  |
| drought | leaf vs stem | 0.13a | 0.97a | (0.07~0.18) | (0.90~1.03) | 0.77 | 0.00 | 196 | crop |
| control | leaf vs stem | 0.12a | 0.96a | (0.06~0.18) | (0.90~1.02) | 0.82 | 0.00 | 196 | crop |
| drought | leaf vs stem | -0.05b | 0.90a | (-0.10~-0.01) | (0.87~0.94) | 0.78 | 0.00 | 470 | wild |
| control | leaf vs stem | -0.04b | 0.89a | (-0.09~0.01) | (0.86~0.94) | 0.76 | 0.00 | 470 | wild |
|  |  |  |  |  |  |  |  |  |  |
| drought | leaf vs stem | -0.12b | 0.90a | (-0.21~-0.02) | (0.81~0.99) | 0.76 | 0.00 | 94 | perennial |
| control | leaf vs stem | -0.07b | 0.91a | (-0.17~0.03) | (0.83~1.00) | 0.79 | 0.00 | 94 | perennial |
| drought | leaf vs stem | 0.02a | 0.94a | (0.04~0.09) | (0.87~1.01) | 0.62 | 0.00 | 295 | annual |
| control | leaf vs stem | 0.01a | 0.90a | (0.06~0.08) | (0.83~0.96) | 0.62 | 0.00 | 295 | annual |
|  |  |  |  |  |  |  |  |  |  |
| drought | repro vs vege | -1.51b | 1.17a | (-2.45~-0.56) | (0.74~1.86) | 0.37 | 0.02 | 15 | woody |
| control | repro vs vege | -2.74b | 1.93a | (-4.45~-1.02) | (1.19~3.11) | 0.31 | 0.03 | 15 | woody |
| drought | repro vs vege | -0.55a | 1.07ba | (-0.66~-0.44) | (0.99~1.16) | 0.61 | 0.00 | 249 | herb |
| control | repro vs vege | -0.56a | 1.09ba | (-0.69~-0.44) | (1.01~1.18) | 0.64 | 0.00 | 249 | herb |
|  |  |  |  |  |  |  |  |  |  |
| drought | repro vs vege | -0.70a | 1.22a | (-0.89~-0.52) | (1.08~1.37) | 0.61 | 0.00 | 110 | crop |
| control | repro vs vege | -0.74a | 1.24a | (-0.97~-0.51) | (1.10~1.40) | 0.60 | 0.00 | 110 | crop |
| drought | repro vs vege | -0.51a | 0.96b | (-0.66~-0.37) | (0.86~1.07) | 0.53 | 0.00 | 154 | wild |
| control | repro vs vege | -0.51a | 0.99b | (-0.66~-0.36) | (0.89~1.09) | 0.58 | 0.00 | 154 | wild |
|  |  |  |  |  |  |  |  |  |  |
| drought | repro vs vege | -0.52a | 0.80b | (-0.81~-0.23) | (0.63~1.00) | 0.67 | 0.00 | 28 | perennial |
| control | repro vs vege | -0.53a | 0.84b | (-0.89~-0.16) | (0.65~1.08) | 0.59 | 0.00 | 28 | perennial |
| drought | repro vs vege | -0.56a | 1.12a | (-0.67~-0.44) | (1.03~1.21) | 0.63 | 0.00 | 221 | annual |
| control | repro vs vege | -0.57a | 1.13a | (-0.69~-0.44) | (1.05~1.22) | 0.67 | 0.00 | 221 | annual |

**Appendix 1**

***Data set 1*** (in an Excel file): Effect size of root mass ratio (RMF), stem mass ratio (SMF), leaf mass ratio (LMF) and reproductive part (ReMF) from 165 studies, combined with related information of plant life forms, cultivation status, experimental condition and treatment duration etc., compiled by Anwar Eziz.

***Data set 2*** (in an Excel file): Plant biomass of root, stem, leaf and reproductive part under well-watered and drought treatments compiled by Anwar Eziz.

***References***

| 1 | Bacelar, E. A., Moutinho-Pereira, J. M., Goncalves, B. C., Ferreira, H. F. and Correia, C. A. (2007) Changes in growth, gas exchange, xylem hydraulic properties and water use efficiency of three olive cultivars under contrasting water availability regimes. *Environmental and Experimental Botany* 60 (2), 183-192. |
| --- | --- |
| 2 | Burslem, D., Grubb, P. J. and Turner, I. M. (1996) Responses to simulated drought and elevated nutrient supply among shade-tolerant tree seedlings of lowland tropical forest in Singapore. *Biotropica* 28 (4), 636-648. |
| 3 | Edwards, C. E., Ewers, B. E. and Weinig, C. (2016) Genotypic variation in biomass allocation in response to field drought has a greater effect on yield than gas exchange or phenology. *Bmc Plant Biology* 16. |
| 4 | Ismail, A. M., Hall, A. E. and Bray, E. A. (1994) Drought and pot size effects on transpiration efficiency and carbon isotope discrimination of cowpea accessions and hybrids. *Australian Journal of Plant Physiology* 21 (1), 23-35. |
| 5 | Luttschwager, D., Ewald, D. and Alia, L. A. (2016) Consequences of moderate drought stress on the net photosynthesis, water-use efficiency and biomass production of three poplar clones. *Acta Physiologiae Plantarum* 38 (1). |
| 6 | Padilla, F. M., Miranda, J. D., Jorquera, M. J. and Pugnaire, F. I. (2009) Variability in amount and frequency of water supply affects roots but not growth of arid shrubs. *Plant Ecology* 204 (2), 261-270. |
| 7 | Sanchez, E., Scordia, D., Lino, G., Arias, C., Cosentino, S. L. and Nogues, S. (2015) Salinity and Water Stress Effects on Biomass Production in Different Arundo donax L. Clones. *Bioenergy Research* 8 (4), 1461-1479. |
| 8 | Susiluoto, S. and Berninger, F. (2007) Interactions between morphological and physiological drought responses in Eucalyptus microtheca. *Silva Fennica* 41 (2), 221-233. |
| 9 | Utkhao, W. and Yingjajaval, S. (2015) Changes in leaf gas exchange and biomass of Eucalyptus camaldulensis in response to increasing drought stress induced by polyethylene glycol. *Trees-Structure and Function* 29 (5), 1581-1592. |
| 10 | Williams, D. G. and Black, R. A. (1994) Drought response of a native and introduced Hawaiian grass. *Oecologia* 97 (4), 512-519. |
| 11 | Yang, Y. Q., Yao, Y., Xu, G. and Li, C. Y. (2005) Growth and physiological responses to drought and elevated Ultraviolet-B in two contrasting populations of Hippophae rhamnoides. *Physiologia Plantarum* 124 (4), 431-440. |
| 12 | Dias, P. C., Araujo, W. L., Moraes, G. A. B. K., Barros, R. S. and DaMatta, F. M. (2007) Morphological and physiological responses of two coffee progenies to soil water availability. *Journal of Plant Physiology* 164 (12), 1639-1647. |
| 13 | Yin, C. Y., Peng, Y. H., Zang, R. G., Zhu, Y. P. and Li, C. Y. (2005) Adaptive responses of Populus kangdingensis to drought stress. *Physiologia Plantarum* 123 (4), 445-451. |
| 14 | Zhang, X., Wu, N. and Li, C. (2005) Physiological and growth responses of Populus davidiana ecotypes to different soil water contents. *Journal of Arid Environments* 60 (4), 567-579. |
| 15 | Zhang, X., Zang, R. and Li, C. (2004) Population differences in physiological and morphological adaptations of Populus davidiana seedlings in response to progressive drought stress. *Plant Science* 166 (3), 791-797. |
| 16 | Marler, T. E. and Clemente, H. S. (2006) Papaya seedling growth response to wind and water deficit is additive. *Hortscience* 41 (1), 96-98. |
| 17 | Guehl, J. M., Picon, C., Aussenac, G. and Gross, P. (1994) Interactive Effects of Elevated CO2 and Soil Drought on Growth and Transpiration Efficiency and Its Determinants in 2 European Forest Tree Species. *Tree Physiology* 14 (7-9), 707-724. |
| 18 | Nash, L. J. and Graves, W. R. (1993) Drought and Flood Stress Effects on Plant Development and Leaf Water Relations of 5 Taxa of Trees Native to Bottomland Habitats. *Journal of the American Society for Horticultural Science* 118 (6), 845-850. |
| 19 | Dale, M. P. and Causton, D. R. (1992) The Ecophysiology of Veronica-chamaedrys, V-montana and V-officinalis .2. the Interaction of Irradiance and Water Regime. *Journal of Ecology* 80 (3), 493-504. |
| 20 | Barrett, D. J. and Ash, J. E. (1992) Growth and Carbon Partitioning in Rain-forest and Eucalypt Forest Species of South Coastal New-south-wales, Australia. *Australian Journal of Botany* 40 (1), 13-25. |
| 21 | Ngugi, M. R., Hunt, M. A., Doley, D., Ryan, P. and Dart, P. (2003) Dry matter production and allocation in Eucalyptus cloeziana and Eucalyptus argophloia seedlings in response to soil water deficits. *New Forests* 26 (2), 187-200. |
| 22 | Pilonsmits, E. A. H., Ebskamp, M. J. M., Paul, M. J., Jeuken, M. J. W., Weisbeek, P. J. and Smeekens, S. C. M. (1995) Improved Performance of Transgenic Fructan-accumulating Tobacco Under Drought Stress. *Plant Physiology* 107 (1), 125-130. |
| 23 | Wendler, R. and Millard, P. (1996) Impacts of water and nitrogen supplies on the physiology, leaf demography and nitrogen dynamics of Betula pendula. *Tree Physiology* 16 (1-2), 153-159. |
| 24 | e Silva, F. C., Shvaleva, A., Maroco, J., Almeida, M., Chaves, M. and Pereira, J. (2004) Responses to water stress in two Eucalyptus globulus clones differing in drought tolerance. *Tree Physiology* 24 (10), 1165-1172. |
| 25 | Egilla, J. N., Davies Jr, F. T. and Drew, M. C. (2001) Effect of potassium on drought resistance of Hibiscus rosa-sinensis cv. Leprechaun: plant growth, leaf macro-and micronutrient content and root longevity. *Plant and Soil* 229 (2), 213-224. |
| 26 | Fang, C. W., Monson, R. K. and Cowling, E. B. (1996) Isoprene emission, photosynthesis, and growth in sweetgum (Liquidambar styraciflua) seedlings exposed to short- and long-term drying cycles. *Tree Physiology* 16 (4), 441-446. |
| 27 | LeRoux, D., Stock, W. D., Bond, W. J. and Maphanga, D. (1996) Dry mass allocation, water use efficiency and delta C-13 in clones of Eucalyptus grandis, E-grandis x camaldulensis and E-grandis x nitens grown under two irrigation regimes. *Tree Physiology* 16 (5), 497-502. |
| 28 | Li, C. (1999) Carbon isotope composition, water-use efficiency and biomass productivity of Eucalyptus microtheca populations under different water supplies. *Plant and Soil* 214 (1-2), 165-171. |
| 29 | Orians, C. M., Bolnick, D. I., Roche, B. M., Fritz, R. S. and Floyd, T. (1999) Water availability alters the relative performance of Salix sericea, Sralix eriocephala, and their F1 hybrids. *Canadian Journal of Botany* 77 (4), 514-522. |
| 30 | Osorio, J., Osorio, M., Chaves, M. and Pereira, J. (1998) Water deficits are more important in delaying growth than in changing patterns of carbon allocation in Eucalyptus globulus. *Tree physiology* 18 (6), 363-373. |
| 31 | Yang, Y., Wang, G. X., Yang, L. D. and Guo, J. Y. (2013) Effects of Drought and Warming on Biomass, Nutrient Allocation, and Oxidative Stress in Abies fabri in Eastern Tibetan Plateau. *Journal of Plant Growth Regulation* 32 (2), 298-306. |
| 32 | Silva, P. E. M., Cavatte, P. C., Morais, L. E., Medina, E. F. and DaMatta, F. M. (2013) The functional divergence of biomass partitioning, carbon gain and water use in Coffea canephora in response to the water supply: Implications for breeding aimed at improving drought tolerance. *Environmental and Experimental Botany* 87, 49-57. |
| 33 | Guenni, O., Marin, D. and Baruch, Z. (2002) Responses to drought of five Brachiaria species. I. Biomass production, leaf growth, root distribution, water use and forage quality. *Plant and Soil* 243 (2), 229-241. |
| 34 | Wu, F. Z., Bao, W. K., Li, F. L. and Wu, N. (2008) Effects of drought stress and N supply on the growth, biomass partitioning and water-use efficiency of Sophora davidii seedlings. *Environmental and Experimental Botany* 63 (1-3), 248-255. |
| 35 | Sun, H. G., Liu, J., Dong, R. X., Jiang, J. M., Diao, S. F. and Yan-Jie, L. I. (2014) Effects of Water Stress on Seedling Growth and Biomass Allocation of Toona ciliata var. pubescens. *Forest Research*. 27, 381-387. |
| 36 | Baruch, Z. (1994) Responses to Drought and Flooding in Tropical Forage Grasses .1. Biomass Allocation, Leaf Growth and Mineral Nutrients. *Plant and Soil* 164 (1), 87-96. |
| 37 | Espinoza, S. E., Martinez, V. A., Magni, C. R., Ivkovic, M., Santelices, R. E., Guerra, F. P. and Cabrera, A. M. (2014) Genetic control of growth, biomass allocation, and survival under drought stress in Pinus radiata D. Don seedlings. *Tree Genetics & Genomes* 10 (4), 1045-1054. |
| 38 | Quezada, I. M. and Gianoli, E. (2010) Counteractive biomass allocation responses to drought and damage in the perennial herb Convolvulus demissus. *Austral Ecology* 35 (5), 544-548. |
| 39 | Liu, F. and Stutzel, H. (2004) Biomass partitioning, specific leaf area, and water use efficiency of vegetable amaranth (Amaranthus spp.) in response to drought stress. *Scientia Horticulturae* 102 (1), 15-27. |
| 40 | Xu, B. C., Li, F. M., Shan, L., Ma, Y. Q., Ichizen, N. and Huang, J. (2006) Gas exchange, biomass partition, and water relationships of three grass seedlings under water stress. *Weed Biology and Management* 6 (2), 79-88. |
| 41 | Paez, A., Gonzalez, M. E., Yrausquin, X., Salazar, A. and Casanova, A. (1995) Water-Stress and Clipping Management Effects on Guineagrass .1. Growth and Biomass Allocation. *Agronomy Journal* 87 (4), 698-706. |
| 42 | Xu, B.-C., Xu, W.-Z., Huang, J., Shan, L. and Li, F.-M. (2011) Biomass allocation, relative competitive ability and water use efficiency of two dominant species in semiarid Loess Plateau under water stress. *Plant Science* 181 (6), 644-651. |
| 43 | Schall, P., Loedige, C., Beck, M. and Ammer, C. (2012) Biomass allocation to roots and shoots is more sensitive to shade and drought in European beech than in Norway spruce seedlings. *Forest Ecology and Management* 266, 246-253. |
| 44 | Li, Y., Qiman, Y. and Zhu, Y. (2006) Effects of water stress on photosynthetic characteristics and biomass partition of Elaeagnus moorcroftii. *Acta Botanica Boreali-Occidentalia Sinica* 26 (12), 2493-2499. |
| 45 | Alvarez, S., Castillo, M., Acosta, J. R., Navarro, A. and Sanchez-Blanco, M. J. (2012) Photosynthetic Response, Biomass Distribution and Water Status Changes in Rhamnus alaternus Plants during Drought. *Xxviii International Horticultural Congress on Science and Horticulture for People (Ihc2010): International Symposium on Advances in Ornamentals, Landscape and Urban Horticulture* 937, 853-860. |
| 46 | Brown, C. E., Mickelbart, M. V. and Jacobs, D. F. (2014) Leaf physiology and biomass allocation of backcross hybrid American chestnut (Castanea dentata) seedlings in response to light and water availability. *Tree Physiology* 34 (12), 1362-1375. |
| 47 | Espinoza, S. E., Magni, C. R., Martinez, V. A. and Ivkovic, M. (2013) The effect of water availability on plastic responses and biomass allocation in early growth traits of Pinus radiata D. Don. *Forest Systems* 22 (1), 3-14. |
| 48 | Xu, B. C., Deng, X. P., Zhang, S. Q. and Shan, L. (2010) Seedling Biomass Partition and Water Use Efficiency of Switchgrass and Milkvetch in Monocultures and Mixtures in Response to Various Water Availabilities. *Environmental Management* 46 (4), 599-609. |
| 49 | Pizarro, L. C. and Bisigato, A. J. (2010) Allocation of biomass and photoassimilates in juvenile plants of six Patagonian species in response to five water supply regimes. *Annals of Botany* 106 (2), 297-307. |
| 50 | Li, F. L., Bao, W. K., Wu, N. and You, C. (2008) Growth, biomass partitioning, and water-use efficiency of a leguminous shrub (Bauhinia faberi var. microphylla) in response to various water availabilities. *New Forests* 36 (1), 53-65. |
| 51 | Erice, G., Louahlia, S., Jose Irigoyen, J., Sanchez-Diaz, M. and Avice, J.-C. (2010) Biomass partitioning, morphology and water status of four alfalfa genotypes submitted to progressive drought and subsequent recovery. *Journal of Plant Physiology* 167 (2), 114-120. |
| 52 | Aranda, I., Alia, R., Ortega, U., Dantas, A. K. and Majada, J. (2010) Intra-specific variability in biomass partitioning and carbon isotopic discrimination under moderate drought stress in seedlings from four Pinus pinaster populations. *Tree Genetics & Genomes* 6 (2), 169-178. |
| 53 | Fernandez, C. J., Cothren, J. T. and McInnes, K. J. (1991) Partitioning of Biomass in Well-watered and Water-stressed Cotton Plants Treated with Mepiquat Chloride. *Crop Science* 31 (5), 1224-1228. |
| 54 | Fernandez, C. J., Cothren, J. T. and McInnes, K. J. (1996) Partitioning of biomass in water- and nitrogen-stressed cotton during pre-bloom. *Journal of Plant Nutrition* 19 (3-4), 595-617. |
| 55 | Achten, W. M. J., Maes, W. H., Reubens, B., Mathijs, E., Singh, V. P., Verchot, L. and Muys, B. (2010) Biomass production and allocation in Jatropha curcas L. seedlings under different levels of drought stress. *Biomass & Bioenergy* 34 (5), 667-676. |
| 56 | Ren, A., Gao, Y., Yu, L., Chen, S., Liu, S. and Liu, N. (1999) Effect of Drought Stress on Clonal Growth of Pennisetum Centrasiaticum and Leymus Secalinus. *Journal of Desert Research* 1, 31-35. |
| 57 | Wang, M., Dai, L. and Ji, L. (2001) A preliminary study on ecological response of dominant tree species in Korean pine broadleaf forest at Changbai Mountain to soil water stress and their biomass allocation. *Chinese Journal of Applied Ecology* 12 (4), 496-500. |
| 58 | Pan, M. (2003) *Growth Plasticity of Endangered Plant Changium Smyrnioides Wolff and Comparative Species to Soil Water*. Master. Hangzhou: Zhejiang University. |
| 59 | Zhao, G. (2015) *The Physiological Response to Drought Stress in Different Provenances of Kapok*. Master. Kunming: Southwest Forestry University of China. |
| 60 | Bai, H., Zhang, R., Zhang, X., Wang, L., Shuang, L. and Jingwen, L. I. (2015) Populus euphratica Seedling Root Growth Characteristics under Different Water Treatment. *Forest Resources Management* 25, 61-66. |
| 61 | Wu, L. (2015) *Response and Adaptation of Cyclobalanopsis Gilva Seedlings to Drought Stress*. Doctor. Changsha, Hunan: Central South University of Forestry Science and Technology of China. |
| 62 | Zhang, Q. (2013) *The Growth and Accumulation of Pb of Amorpha Fruticosa and Platycladus Orientalis under Water and Lead Stresses*. Master. Yangling, Shanxi, China: Northwest A & F University of China. |
| 63 | Wang, Q., Liu, X., Wang, H., Meng, X., Cao, G., Li, J. and Huang, C. (2015) Effects of drought and waterlogging on growth and photosynthesisof potted young Pinus tabulaeformis Carr. *Science of Soil and Water Conservation of China* 6, 40-47. |
| 64 | Wang, Y.-l., Xu, Z.-z. and Zhou, G.-S. (2004) Changes in biomass allocation and gas exchange characteristics of Leymus chinensis in response to soil water stress. *Acta Phytoecologica Sinica* 28 (6), 803-809. |
| 65 | Yang, Z. Z., Ma, Y. Y., Mi, X. Q., Yang, Y., Jin, L. X. and Zong, L. (2011) Effect of organs biomass by drought stress on the leaf growing stage of Angelica dahurica var. formosana. *Journal of Chinese Medicinal Materials* 34 (4), 503-506. |
| 66 | Jia, R., Yang, Z., Daping, X. U., Zhang, N. and Liu, X. (2013) Growth and endogenous hormone content of Dalbergia odorifera seedlings under drought stress. *Ecology & Environmental Sciences* 22 (7), 1136-1140. |
| 67 | Wu, C., Jiang, C., Xie, P., He, Y. and Yang, J. (2015) Effects of Campora Seedlings on Biomass Allocation and Growth under Drought Stress and Inoculated AMF. *Journal of Chongqing Normal University (Natural Science) of China* 6, 109-115. |
| 68 | Long, J. (2013) *Effects of Soil Drought on Non-structural Carbon and Nitrogen Contents of Different Organs and Long-distance Transportation in Three Species Woody Plants*. Master. Yangling, Shanxi: Northwest A & F University of China. |
| 69 | Yan, H. X., Fang, L. B. and Huang, D. Z. (2011) Effects of drought stress on the biomass distribution and photosynthetic characteristics of cluster mulberry. *Chinese Journal of Applied Ecology* 22 (12), 3365-3370. |
| 70 | Zhang, W. and Farm, S. F. (2016) Effects of Drought Stress on Biomass Allocation of Ulmus pumila Seedlings. *Protection Forest Science & Technology* 9, 27-29. |
| 71 | Zhang, Y. M., Ma, K. M., Li, F. L. and Qu, L. Y. (2016) Arbuscular mycorrhizal fungi (AMF) promotes Bauhinia faberi var. microphylla seedling growth under drought stress conditions. *Acta Ecologica Sinica* 11, 3329-3337. |
| 72 | Yue, Y. (2012) *Growth and Physiological Biochemical Characteristics of Seedling in Different Myrica Rubra Species under Drought Stress*. Master. Lin'an, Zhejiang,China: Zhejiang A&F University. |
| 73 | Liu, P. (2011) *Studies on the Seedling Physiological-biochemical Characteristics in Different Camptotheca Acuminata Provenance under Drought Stress*. Master. Lin'an, Zhejiang,China: Zhejiang Agriculture and Forestry University. |
| 74 | Qiu, Q., Ji-Yue, L. I., Wang, J. H., Qian, H. E., Yan, S. U., Jian-Wei, M. A., Kun, D. U. and Pan, X. (2013) Biomass and Root System Characteristics and Drought Resistance of 4 Shrubs in Tibetan Plateau under Drought Stress. *Journal of Northwest Forestry University* 3 (33), 1-6. |
| 75 | Wu, Y. (2013) *Effects of environmental factors on physiology, biochemistry and chemical composition of Polygala tenuifolia*. Master. Northeast University of China. |
| 76 | Yan, H. (2015) *Studies on The Physiological Mechanism of The Stress Adaptability of Flaveria Bidentis(L.) Kuntze*. Doctor. Hebei Agricultural University of China. |
| 77 | Hu, H. (2012) *Water Consumption Characteristic and Response to Drought Stress of Eucalyptus Grandis*. Doctor. Ya'an, Sichuan: Sichuna Agricultural University of China. |
| 78 | Wang, R. (2011) *Effects of Mycorrhizal Fungal on Growth Status of Seedings in Karst Areas*. Master. Nanjing Forestry University of China. |
| 79 | Zhao, J. (2013) *Roots Characteristics of Populus×euramericana cv.’Neva’ and Effects of Salt Stress and Drought Stress on Physiological Characteristics*. Master. Shandong Agricultural University. |
| 80 | Zhang, N., Yang, X., Cao, D., Jingwen, L. I., Jing, J., Shuang, L. and Xia, Y. (2013) Soil Water and Fertilizer Factors on the Trade-off of Growth and Lignification of Populus euphratica Seedling. *Acta Botanica Boreali-Occidentalia Sinica* 33 (4), 771-779. |
| 81 | Li, Z., Gao, K. M., Liu, J. C., Liang, Q. H. and Tao, J. P. (2016) Growth response of two annual herb species to alternating drying-wetting and nitrogen addition in the karst area of Southwest China. *Acta Ecologica Sinica* 11, 3372-3380. |
| 82 | Song, L. N., Zhu, J. J. and Kang, H. Z. (2013) Response of Hydraulic Structure Parameters and Growth of Pinus sylvestris var. mongolica Seedling to Simulated Precipitation Gradient. *Arid Zone Research* 6 1021-1027. |
| 83 | Li, T. (2011) *The Water Physio-ecology Mechanism of Hippophae Rhamnoides L. Subsp. Sinensis Decline Plantation*. Doctor. Beijing Forestry University. |
| 84 | Zhang, K. (2016) *The Resource Use Efficiencies And Trade off among Spring Wheat Cultivars with Different Drought Resistance*. Doctor. Gansu Agricultural University of China. |
| 85 | Duan, G. (2016) *Effects of simulated precipitation pattern on seed germination and seedling growth of Reaumuria songarica*. Master. Gansu Agricultural University of China. |
| 86 | Xu, F., Guo, W. H., Xu, W. H. and Wang, R. Q. (2010) Effects of water stress on morphology, biomass allocation and photosynthesis in Robinia pseudoacacia seedlings. *Journal of Beijing Forestry University* 32 (1), 24-30. |
| 87 | Liu, J. P. and Duan, J. (2013) Humulus scandens gender differences in response to water stress in the vegetative growth stage. *Acta Prataculturae Sinica* 22 (2), 243-249. |
| 88 | Zhang, C. (2003) *Ecophysiologcial Responses to Soil Drought in Four Woody Species Seedling in Quercus Liaotungensis Forest*. Doctor. Northeast Forestry University of China. |
| 89 | Yuyan, A. N. and Liang, Z. (2011) Growth and physiological responses of the Periploca sepium Bunge seedlings to drought stress. *Acta Ecologica Sinica* 31 (3), 716-725. |
| 90 | Sun, Z. (2002) *A Study on the Drought Resistance of Acer ginnala, Malus Baccata, Prunus Davidiana and Pyrus Ussuriensis*. Master. Northeast Forestry University of China. |
| 91 | Deng, Y., Wang, B., Su, W. H., Zhang, G. F. and Deng, X. B. (2010) Phenotypic plasticity and physiological responses of Eucalyptus grandis × E. urophylla seedling under drought stress. *Acta Botanica Boreali-Occidentalia Sinica* 6, 1173-1179. |
| 92 | Chen, M. T. (2011) Effects of drought on root characteristics and mass allocation in each part of seedlings of four tree species. *Journal of Beijing Forestry University* 33 (1), 16-22. |
| 93 | Huang, X. X., Yang, Z. Y., Gao, K. and Cheng, X. M. (2011) Growth and Physiological Responses to Soil Drought and Salt Stress in Itoa orientalis Seedlings. *Southwest China Journal of Agricultural Sciences* 24 (3), 896-900. |
| 94 | Chen, X. and Yi-Feng, X. U. (2011) Effects of drought stress on growth and physiological characteristics in Sorbus folgneri seedlings. *Journal of Fujian College of Forestry* 31 (4), 330-334. |
| 95 | Liu, Y., Chen, G. L., Cai, G. F., Zhang, Z. X. and Yue, X. (2011) Growth and Osmoregulation Substances Accumulation of Glycyrrhiza uralensis Seedling under Drought Stress. *Acta Botanica Boreali-Occidentalia Sinica* 31 (11), 2259-2264. |
| 96 | Miao, F., Luo, J., Wang, H., Yang, B. and Wang, L. (2015) Drought Resistance of Potato Seedlings and Effects of Phosphorus and Potassium Fertilizer on Their Drought Resistance. *Jiangsu Agricultural Sciences of China* 5, 92-94. |
| 97 | Yang, W., Sun, Z. and Wang, Q. (2002) Effects of Different Soil Water Content on Rooting and Seedling Growth of Populus alba×Populus berolinensis Cutting. *Journal of Northeast Forestry University* 30 (4), 125-128. |
| 98 | Shan, L., Li, Y., Shi, W. and Li, S. (2015) Effects of Dehydration Stress on Growth of Reaumuria Soongorica Seedlings and Regulation of Osmotic Substances. *Bulletin of Soil and Water Conservation* 6, 106-109. |
| 99 | Dong-Qin, L. I., Zeng, P. C., Chen, G. K. and Hua-Shou, L. I. (2016) Effects of drought stress on biomass distribution and physiological characteristics in three kinds of leguminous shrubs. *Journal of Central South University of Forestry & Technology* 1, 33-39. |
| 100 | Wu, Y., Linghong, W., Dongliang, C., Xu, C., Zhang, Z., Li, J. and Zhong, Q. (2016) Effect of water and nitrogen treatment on photosynthetic characteristics and the biomass allocation of annual cutting seedling of Machilus pauhoi. *Journal of Anhui Agricultural University* 2, 202-208. |
| 101 | Zhou, F. (2013) Interaction of Drought and Pb on Growth and Antioxidant Enzyme Activities of Platycladus orientalis Seedlings. *Scientia Silvae Sinicae* 49 (6), 172-177. |
| 102 | Zhao, J. H., Li, H. X., Zhou, X., An, W., Shi, Z. G., Wang, Y. J. and Wang, H. F. (2012) Influence of drought stress on plant growth and sugar accumulation in fruit of Lycium barbarum L. *Plant Physiology Journal* 48 (11), 1063-1068. |
| 103 | Rong, H., Xia, L. I. and Ren, A. (2011) Physiological ecological effect of endophyte infection on Achnatherum sibiricum under drought stress. *Acta Ecologica Sinica* 31 (8), 2115-2123. |
| 104 | Huang, X. X., Shao-Bo, H. U. and Deng, L. L. (2011) Growth and Physiological Traits of Protea cynaroides Cuttings as Affected by Soil Drought and Salt Stress. *Northern Horticulture* 15, 97-100. |
| 105 | Wang, X. D., Cao, L. P. and Liu, Y. D. (2011) Effects of Drought Stress on Root Morphology & Biomass Allocation of Betula maximowicziana. *Protection Forest Science & Technology* 5 (50), 20-22. |
| 106 | Yang, H., Tang, Q., Hang, Y. and Wang, X. P. (2010) Eco-physiological response of Mingshanbaihao tea seedle to drought stress. *Southwest China Journal of Agricultural Sciences* 23 (5), 1497-1503. |
| 107 | Yang, Z., Chaoying, M., Xiaoqin, M., Yang, B. and Jin, L. (2010) Effects of Drought Stress on Vegetative Organs biomass and growth of Angelica dahurica. *Chinese Medicine Society of the 10th session of the Chinese Medicine Identification Conference.* |
| 108 | Xi, M. (2010) *Effect of Drought Stress on Anatomical Structure and Physiological Characteristics in Solanum Nigrum*. Master. Nanjing Agricultural College of China. |
| 109 | Zhao, Q. (2015) *Effect of Elevated Temperature and Drought Stress on the Growth and Fruit Quality of Lycium Bararum*. Master. Ningxia University of China. |
| 110 | Yang, T. (2015) *Induction of Root-shoot Signals and Regulation of Yield Formation by Root Partition Irrigation in Spring Wheat*. Master. Lanzhou University of China. |
| 111 | He, S. (2015) *The Repair of Heavy Metal Pollution of Cadmium and Characteristics of Drought on Puelia Sinese*. Master. Sichuan Agricultural University of China. |
| 112 | Li, Q. (2013) *Physiological Responds and Adaptation of Miscanthus Sacchariflorus and Miscanthus Sinensis to Drought Stress*. Doctor. Northeast Forestry University of China. |
| 113 | Qin, J. (2011) *Drought and Salinity Resistant Physiology and Water Consumption Characteristics of Main Plantation Tree Species in High-cold Region of Loess Plateau*. Doctor. Beijing Forestry University of China. |
| 114 | Cang, Z. (2011) *Diversity Analysis of the Floral Traits and Study on the Drought Resistance of Magnolia Wufengensis*. Doctor. Beijing Forestry University. |
| 115 | Zhao, S. (2013) *Study on Water Factor Influence on Yield and Quality of Scutellaria Baicalensis*. Master. Jilin Agricultural University of China. |
| 116 | Wu, Y., Wang, L., Cheng, D., Xu, C., Zhang, Z., Li, J. and Zhong, Q. (2016) Effect of water and nitrogen treatment on photosynthetic characteristics and the biomass allocation of annual cutting seedling of Machilus pauhoi. *Journal of Anhui Agricultural University of China China* 43 (2), 202-208. |
| 117 | Li, H., Zeng, P., Chen, G. and Li, H. (2016) Effects of drought stress on biomass distribution and physiological characteristics in three kinds of leguminous shrubs. *Journal of Central South University of Forestry & Technology* 36 (1), 33-39. |
| 118 | Dong, X., Patton, J., Wang, G., Nyren, P. and Peterson, P. (2014) Effect of drought on biomass allocation in two invasive and two native grass species dominating the mixed-grass prairie. *Grass and Forage Science* 69 (1), 160-166. |
| 119 | Wang, Y.-l., Xu, Z.-z. and Zhou, G.-S. (2004) Changes in biomass allocation and gas exchange characteristics of Leymus chinensis in response to soil water stress. *Acta Phytoecologica Sinica* 28 (6), 803-809. |
| 120 | Lu, H., Ma, X., Zhang, R., Zhong, X. L., Zhu, C. G. and Yang, Y. H. (2016) Effects of different forms of nitrogen on the growth and physiology of Tamarix ramosissimaseedlings under water stress. *Acta Prataculturae Sinica*. |
| 121 | Chen, X., Tan, J. D., Ding, Y. L., Xuan, Z. Y. and Huan, K. E. (2016) Effects of Drought Stress on Growth of Carallia brachiata Seedling. *Journal of Fujian Forestry Science & Technology* 2, 129-133. |
| 122 | Zhang, Y. M., Ma, K. M., Li, F. L. and Qu, L. Y. (2016) Arbuscular mycorrhizal fungi (AMF) promotes Bauhinia faberi var. microphylla seedling growth under drought stress conditions. *Acta Ecologica Sinica* 11, 3329-3337. |
| 123 | Mao, Y., Liu, L. and Wang, X. (2016) Effect of drought stress on physiological characteristics of three plants of Aceraceae. *Journal of Zhejiang A & F University* 33 (1), 60-64. |
| 124 | Huang, Y. X., Zhao, X. Y., Zhou, D. W., Wang, T. H., Li, G. D. and Li, Q. (2013) Biomass Allocation to Vegetative and Reproductive Organs of Chenopodium Acuminatum Willd. Under Soil Nutrient and Water Stress. *Bangladesh Journal of Botany* 42 (1), 113-121. |
| 125 | Ge, T. D., Sui, F. G., Bai, L. P., Tong, C. L. and Sun, N. B. (2012) Effects of water stress on growth, biomass partitioning, and water-use efficiency in summer maize (Zea mays L.) throughout the growth cycle. *Acta Physiologiae Plantarum* 34 (3), 1043-1053. |
| 126 | Mahieu, S., Germon, F., Aveline, A., Hauggaard-Nielsen, H., Ambus, P. and Jensen, E. S. (2009) The influence of water stress on biomass and N accumulation, N partitioning between above and below ground parts and on N rhizodeposition during reproductive growth of pea (Pisum sativum L.). *Soil Biology & Biochemistry* 41 (2), 380-387. |
| 127 | Greco, S. A. and Cavagnaro, J. B. (2003) Effects of drought in biomass production and allocation in three varieties of Trichloris crinita P. (Poaceae) a forage grass from the arid Monte region of Argentina. *Plant Ecology* 164 (1), 125-135. |
| 128 | Xia, M. Z. (1997) Effects of soil drought during the generative development phase on seed yield and nutrient uptake of faba bean (Vicia faba). *Australian Journal of Agricultural Research* 48 (4), 447-451. |
| 129 | Moolman, A. C., vanRooyen, N. and vanRooyen, M. W. (1996) The effect of drought stress on the dry matter production, growth rate and biomass allocation of Anthephora pubescens Nees. *South African Journal of Botany* 62 (1), 41-45. |
| 130 | Rozijn, N. and Vanderwerf, D. C. (1986) Effect of Drought During Different Stages in the Life-cycle on the Growth and Biomass Allocation of 2 Aira Species. *Journal of Ecology* 74 (2), 507-523. |
| 131 | Sobrado, M. A. and Turner, N. C. (1986) Photosynthesis, Dry-matter Accumulation and Distribution in the Wild Sunflower Helianthus petiolaris and the Cultivated Sunflower Helianthus annuus as Influenced by Water Deficits. *Oecologia* 69 (2), 181-187. |
| 132 | Liu, J., You, M., Duan, J. and Zhang, L. (2015) Plasticity of reproductive strategy of dioecious Humulusscandensin response to variation in water deficit stress. *Acta Prataculturae Sinica*. |
| 133 | Liu, J. (2014) *Effects of Water Condition on the Biomass Allocation and Yield Formation of Naked Oats*. Master. Lanzhou University. |
| 134 | Sun, H. (2014) *Toxin Biosynthesis, Physiological Plasticity and Carbon Assimilation Under the Operation of Root-sourced Signal in Grass Pea (Lathyrus Sativus L.)*. Doctor. Lanzhou University Thesis. |
| 135 | Cai, L. (2012) *The Mechanism of Eco-physiological Respond to Enviromental Stress for Pioneer Plant Neyraudia Reynaudiana in Collapsing Hill Area*. Doctor. Fujian Agriculture and Forestry University of China. |
| 136 | Zhang, X. (2011) *Differentiate Expression of Drought Induced Protein and Biomass Partition Pattern in Response to Root-sourced Signal Regulation in Wheat Crop*. Master. Lanzhou University. |
| 137 | Xiong, J. (2012) *Eco-physiological Effect of24-epibrassinolide on β-ODAP Biosynthesis and Drought Adaptability and Its Mechanism in Grass Pea (Lathyrus Sativus L.)*. Master. Lanzhou University Thesis. |
| 138 | Yang, S., Wang, B. and Xiao, X. Sex-specific Responses of Flowering Phenology and Floral Morphology of Humulus scandens to Drought. *Plant Diversity and Resources of China* 5, 653-660. |
| 139 | Li, J. (2002) *Ecological Adaptation Strategies of Dominant Species in Inner Mongolia Typical Steppe*. Doctor. Gansu University Thesis. |
| 140 | Guan, B. (2004) *Comparative Study on Phenotypic Plasticity of Four Mosla Species in Response to Soil Water Status*. Doctor. Zhejiang University. |
| 141 | Geng, Y. (2006) *Adaptive Strategies of Invasive Alligator Weed, Alternanthera Philoxeroides, in Heterogeneous Habitats*. Doctor. Fudan University: |
| 142 | Li, F. (2007) *Acclimation and Adaptation of Three Leguminous Shrub Species to Drought Stress*. Doctor. Chengdu Institute of Biology: Chinese Academy of Sciences. |
| 143 | Chen, S. (2001) *Study on Root/Shoot Growth and Their Relations under Fluctuated Soil Moisture in Winter Wheat Plant*. Post Doctor. Chinese Academy of Agricultural Sciences. |
| 144 | Sun, Y. (2007) *Influences of Water Stress on Growth and Development, Physiological Characteristics and Nutrient Distribution of Winter Wheat*. Master. Chinese Academy of Agricultural Sciences. |
| 145 | Jin, J. (2005) *Effect of Water Treatment to Growth and Reproduction of Cajanus Cajan (L) Millspaugh in the Limestone Zone*. Master. Southwest Normal University. |
| 146 | Guan, B., Ying, G., Fan, M., Niu, X., Lu, Y. and Jie, C. (2003) Phenotypic plasticity of growth and morphology in Mosla chinensis responds to diverse relative soil water content. *Acta Ecologica Sinica* 23 (2), 259-263. |
| 147 | He, J. (2013) *The Non-hydraulic Root Sourced Signal and Osmotic Adjustment in Related to the Yield Performance in Soybean under Drought Condition*. Master. Lanzhou University. |
| 148 | Gu, Y., Ding, S., Gao, Z. and Xing, Q. (2012) Influence of drought and rewatering on the pattern of photosynthate partitioning of winter wheat. *Engineering Sciences*. |
| 149 | Huang, Y., Zhao, X., Zhang, H., Japhet, W., Zuo, X., Luo, Y. and Huang, G. (2009) Allometric effects of Agriophyllum squarrosum in response to soil nutrients, water, and population density in the Horqin Sandy Land of China. *Journal of Plant Biology* 52 (3), 210-219. |
| 150 | Penuelas, J., Biel, C. and Estiarte, M. (1993) Changes in biomass, chlorophyll content and gas exchange of beans and peppers under nitrogen and water stress. *Photosynthetica* 29 (4), 535-542. |
| 151 | Sultan, S. and Bazzaz, F. (1993) Phenotypic plasticity in Polygonum persicaria. II. Norms of reaction to soil moisture and the maintenance of genetic diversity. *Evolution*, 1032-1049. |
| 152 | Aronson, J., Kigel, J. and Shmida, A. (1993) Reproductive Allocation Strategies in Desert and Mediterranean Populations of Annual Plants Grown with and without Water-stress. *Oecologia* 93 (3), 336-342. |
| 153 | Chipman, R. B., Raper, C. D. and Patterson, R. P. (2001) Allocation of nitrogen and dry matter for two soybean genotypes in response to water stress during reproductive growth. *Journal of Plant Nutrition* 24 (6), 873-884. |
| 154 | Wu, X. and Bao, W. (2011) Influence of water deficit and genotype on photosynthetic activity, dry mass partitioning and grain yield changes of winter wheat. *African Journal of Agricultural Research* 6 (25), 5567-5574. |
| 155 | Baigorri, H., Antolin, M. C. and Sanchez-Diaz, M. (1999) Reproductive response of two morphologically different pea cultivars to drought. *European Journal of Agronomy* 10 (2), 119-128. |
| 156 | Kumar, A. and Sharma, K. (2009) Physiological responses and dry matter partitioning of summer mungbean (Vigna radiata L.) genotypes subjected to drought conditions. *Journal of Agronomy and Crop science* 195 (4), 270-277. |
| 157 | Purcell, L. C., deSilva, M., King, C. A. and Kim, W. H. (1997) Biomass accumulation and allocation in soybean associated with genotypic differences in tolerance of nitrogen fixation to water deficits. *Plant and Soil* 196 (1), 101-113. |
| 158 | Sanchez-Blanco, M. J., Alvarez, S., Navarro, A. and Banon, S. (2009) Changes in leaf water relations, gas exchange, growth and flowering quality in potted geranium plants irrigated with different water regimes. *Journal of Plant Physiology* 166 (5), 467-476. |
| 159 | Yanfang, G. U., Ding, S. and Gao, Z. (2010) The pattern of photosynthate partitioning in drought-stressed winter wheat and its relationship with yield. *Acta Ecologica Sinica* 30 (5), 1167-1173. |
| 160 | Chen, X. and Luo, Y. (2002) Compensatory effects of water-recovery during different growth durations on winter wheat under water stress. *Chinese Journal of Eco-Agriculture* 10 (1), 35-37. (In Chinese) |
| 161 | Zhao, L., Deng, X. and Shan, L. (2002) Compensatory effect of varied water conditions on spring wheat before and after flowering. *Chinese Journal of Applied & Environmental Biology* 8 (5), 478-481. (In Chinese) |
| 162 | Kang, L. and Wei, Y. (1995) Effects of Soil Moisture on Dry Mass of Winter Wheat and Distribution of Nutrient Elements under Different Nitrogenous Fertilizers. *Research of Soil and Water Conservation of China* 2 (1), 27-30. (In Chinese) |
| 163 | Hu, J., Cao, W., Jiang, D. and Luo, W. (2004) Quantification of water stress factor for crop growth simulation I. Effects of drought and waterlogging stress on photosynthesis, transpiration and dry matter partitioning in winter wheat. *Acta Agronomica Sinica* 30 (4), 315-320. (In Chinese) |
| 164 | Fang, X., Turner, N. C., Yan, G., Li, F. and Siddique, K. H. (2009) Flower numbers, pod production, pollen viability, and pistil function are reduced and flower and pod abortion increased in chickpea (Cicer arietinum L.) under terminal drought. *Journal of experimental botany*, erp307. |
